# Participants’ perspectives of the advanced ovarian cancer biomarker study VALTIVE1: a qualitative study

**DOI:** 10.1136/bmjopen-2024-088474

**Published:** 2025-07-13

**Authors:** Daniella Holland-Hart, Margherita Carucci, Magdalena Slusarczyk, Mirella Longo, Susan Campbell, Alys Irving, Simon Noble, Gordon Jayson, Noreen Hopewell-Kelly

**Affiliations:** 1 Cardiff University, Cardiff, UK; 2 The Christie NHS Foundation Trust, Manchester, UK

**Keywords:** ONCOLOGY, Patient-Centered Care, QUALITATIVE RESEARCH, Gynaecological oncology

## Abstract

**Objectives:**

VALTIVE1 is a multi-centre, single-arm, non-interventional biomarker study for patients with advanced ovarian cancer. Plasma samples (Tie2 concentration) are collected to detect vascular control in tumours during standard treatment with chemotherapy and bevacizumab. This qualitative study embedded in VALTIVE1 aimed to assess the acceptability and feasibility of a potential VALTIVE2 trial. It explored the participants’ perceptions of the study and treatments and how they might feel if bevacizumab were discontinued based on the results from the biomarker test.

**Design:**

This qualitative study used semi-structured telephone interviews, which were analysed using deductive and inductive thematic analysis.

**Settings:**

Cancer treatment sites in the UK.

**Participants:**

Participants recruited to VALTIVE1 were invited to take part in qualitative interviews. 11 female participants took part from four clinical sites.

**Results:**

Participants reported that they experienced side effects attributed to bevacizumab, including stiffness, pain, fatigue, nose bleeds and muscle aches. Participants felt that combining chemotherapy and bevacizumab may have increased the severity of the side effects they experienced. Most participants felt that it was acceptable, if not preferable, to be allocated to a group in a future VALTIVE2 study where bevacizumab may be discontinued according to the results from the biomarker test. A clear preference of participants was to be informed of the biomarker test results, health status and treatment side effects.

**Conclusion:**

A future trial should consider ensuring all participants have access to test results, as participants indicated a preference to know whether bevacizumab was working and to discontinue bevacizumab if it had not prevented tumour growth based on the biomarker results. Comprehensive and ongoing information and support regarding treatment side effects should be provided to all participants throughout their cancer pathways and trials.

**Trial registration number:**

NCT04523116.

**STRENGTHS AND LIMITATIONS OF THIS STUDY**

- This qualitative study highlights in-depth, nuanced participants’ experiences of standard treatment for advanced ovarian cancer (chemotherapy and bevacizumab) and the VALTIVE1 study.
- Interviews illustrate the side effects and psychological impact of treatment on participants, and their attitudes towards discontinuation of bevacizumab.
- Qualitative data analysis and real-time reporting occurred throughout the study, informing the VALTIVE1 team of potential improvements to VALTIVE1 and a future VALTIVE2 study.
- Significant delays to essential permissions for qualitative researchers from clinical sites slowed down and reduced opportunities for participant interviews.
- No participants were able to be interviewed before treatment, only after treatment began, which potentially reduced their ability to accurately recall their experiences at the start of VALTIVE1.

## Background

 Ovarian cancer (OC) has a poor prognosis with around a 45% 5-year survival rate, due to most cases being diagnosed at an advanced stage.^1^ Bevacizumab is a targeted systemic anticancer therapy which inhibits vascular endothelial growth factor. When used in addition to chemotherapy, it increases progression-free survival and overall survival in advanced OC.^2^,^6^ However, bevacizumab has an established side-effect profile, making combination therapy more onerous than chemotherapy therapy alone. Using a biomarker to identify patients most likely to respond to bevacizumab could have a significant impact on clinical practice, quality of life and health economics.

The VALTIVE research programme explores the utility of the protein Tie2, as a biomarker of response to bevacizumab. VALTIVE1 is a multi-centre, non-randomised observational study of patients with stage IIIc/IV OC receiving first-line platinum-based chemotherapy and bevacizumab, in which blood samples are collected for the Tie2 test. VALTIVE1 examines whether patients whose Tie2 (biomarker) level decreases in response to bevacizumab will have OC that is controlled for much longer than those where the Tie2 level does not decrease. This non-intervention study involves standard treatment doses and schedules of bevacizumab. VALTIVE1 requires extra plasma sample acquisition (at the same time as bevacizumab or chemotherapy is administered) and monitoring of the patient’s condition. The blood sample schedule is available in the protocol.^7^ The results of VALTIVE1, including this qualitative study, will influence the design of a subsequent VALTIVE2 trial, which will aim to establish conclusively the utility of the Tie2 test.

The side-effect profile of bevacizumab is well established^8^ and clinical trials have evaluated this from a safety perspective using standard EORTC toxicity grading,^9^ yet they offer little insight into the patients’ experience of the drug, including toxicities, which may have a profound impact on their quality of life.

Qualitative methods have been used in previous trials to explore participants’ experiences and perceptions of treatments and inform trial conduct.^10^,^13^ These have offered in-depth perspectives beyond side-effect profiles, as they contextualise the treatment regimen within an individual’s lived experience. Such data also provide insights into treatment practicalities and compliance. However, earlier OC studies exploring how side effects from combined chemotherapy and bevacizumab impact quality of life have not included patient perspectives using qualitative methods.^214^,^16^ We therefore carried out a qualitative sub-study to develop an in-depth understanding of patients’ experiences of participating in the VALTIVE1 study.

### Aims and objectives

The aim of this qualitative interview study within VALTIVE1 was to inform the design of the future randomised trial (VALTIVE2). It sought to explore nuanced participants’ experiences of the VALTIVE1 study and treatments, and how these impacted their quality of life, rather than generalisability for those experiencing OC. This included exploring how they might feel if their treatment were to be discontinued based on the results of a biomarker test.

The main objectives of the qualitative study were to:

1. Ascertain whether patients can differentiate between bevacizumab or other chemotherapy side effects.
2. Consider the trade-off to be made between side effects and treatment in advanced disease.
3. Assess the severity of side effects from bevacizumab.
4. Check understanding of randomisation.
5. Assess the acceptability of treatment allocation where treatment may be discontinued.
6. Identify relevant patient-centred outcomes for the subsequent trial and consider time points for assessment.

## Methods

This qualitative sub-study of VALTIVE1 used interview methods to explore participants’ views of the main study and treatments. Data were used to inform the future trial design and optimise recruitment. 

### Public and patient involvement (PPI)

The Trial Management Group (TMG) was supported by two research partners (patient representatives) who contributed to the design and provided general oversight of VALTIVE1, reviewed study documentation, contributed to TMG meetings, and evaluation and contextualisation of the qualitative study results. Both research partners are members of the VALTIVE1 Study Management Group.

### Participants

A subset of VALTIVE1 participants was invited to participate in interviews, with the aim of recruiting 10–20 participants. Interview participants were recruited from four of the five sites which signed up for the qualitative trial out of nineteen sites participating in VALTIVE1. Participants were aged 16 years or older and had FIGO stage IIIc/IV OC on treatment with first-line platinum-based chemotherapy and bevacizumab.

### Recruitment

The main VALTIVE1 study commenced recruitment on 31 March 2021 with a planned completion date of August 2025. Participants in the qualitative sub-study were recruited between September 2022 and October 2023. Potential participants were provided with information about the VALTIVE1 study and the qualitative study via written Participant Information Sheets (PISs), accompanied by an oral explanation provided by recruiting teams. All trial staff received site initiation training, which also covered how to explain the VALTIVE1 and the qualitative study to participants. The PIS was written in plain language and checked by the patient and public involvement representative to ensure it was user-friendly for participants. VALTIVE1 participants were provided with contact details of the trial staff in case they required further information about the study. Language translators and interpreters were provided to patients on request. Participants were not offered payment for their participation in the study and were self-selecting.

Interview participants were recruited through the following process:

1. Recruiting teams at clinical sites distributed the qualitative study PIS, consent form and consent to contact form to potential participants. Interested participants sent signed consent to contact forms or consent forms (in a prepaid envelope) to the qualitative researchers. Once participants consented to VALTIVE1 and the qualitative study, they could be interviewed at any time point during their participation in VALTIVE1.
2. The qualitative team contacted the participant (via email, telephone or letter) to organise a suitable time for the interview.
3. Consent was taken via telephone at the time of the interview, unless participants had provided hard copy consent forms. Telephone consent was appropriate as COVID-19 was still a concern, so it reduced the need for face-to-face contact and the time required for postage. Consent was audio-recorded, and each consent question was read aloud by the researcher and agreed on by the participant. The consent form was signed on behalf of the participant, and a copy was sent to them. Digital copies of the consent form were stored separately from the audio recordings and transcripts.

The recruitment process was complicated by delays, as permissions to conduct interviews could not take place until letters of access from individual study sites were issued. Consequently, most participants were contacted by the study team several months after consenting to the main study. 11 potential participants who signed the consent to contact forms or consent forms were unable to be interviewed as the recruitment process was impeded by complications. The reasons for non-participation include: one participant withdrew from the study prior to the interview, three study participants signed the consent to contact forms but they did not respond during follow-up, and three contact forms were signed, but letters of access from their sites were not received in time for interviews to be conducted. Also, four participants signed consent forms, but their contact details were not sent to the qualitative researchers, so they were not able to be contacted.

### Data collection

Semi-structured interviews were conducted by telephone by two female qualitative post-doctoral researchers (lead and senior authors). They have over 10 years each of experience in exploring participants’ trial experiences, and sensitive health-related topics including cancer. The qualitative researchers had no prior relationship to the participants and used their research experience and training in qualitative methods to introduce the research study and mitigate the asymmetry of information between the two parties. They were not involved in participants’ treatment or any other part of the main study, and therefore, were able to critically explore elements of participants’ experiences of VALTIVE1. Before the interviews began, participants were made aware of the researchers’ roles, university and department, the purpose of the study, and were provided with the opportunity to ask any questions.

Semi-structured interview schedules were used to ensure all the main topics were covered across the interviews, but also allowed for discussions to be guided by the participants. Interview schedules were tested by a senior qualitative researcher (Annmarie Nelson) and checked by the PPI for suitability. There were two sets of questions: one for baseline interviewees (after signing up to the trial and before treatment) and one for those after initial treatment. Participants were unable to be interviewed at baseline, so all participants were asked questions about being recruited to the study during interviews after their treatment started. Interview topics included experiences of recruitment to the study; treatment experiences; impact of treatment on quality of life; impact of the coronavirus pandemic and accessing other services, and views on VALTIVE2. Interview schedules are available in online supplemental file 1, this includes a visual representation prompt available to the interviewer to help explain the potential randomised control VALTIVE2 trial to participants. General notes were made of the interview for the researchers’ use. All audio recordings were transcribed verbatim by a transcription company after a confidentiality agreement was signed by the company and Cardiff University.

### Data analysis

Data were analysed using both deductive and thematic inductive analysis. All the transcripts were originally coded by the lead author and 50% were double-coded by the senior author. Initially, broad themes were captured by analysing interview data deductively, and key findings were developed in accordance with the qualitative study’s objectives.^17^ Synchronously, an inductive thematic analysis was conducted.^18^ Initially, researchers coded transcripts separately, then agreed on a coding structure together, including all the main themes and their concomitant categories. Themes were generated by combining relevant codes and identifying overarching concepts. The coding structure and analysis framework were refined as an iterative process, final codes and themes were agreed on through ongoing deliberations. A codebook was then established to organise the themes and subthemes, which both researchers agreed on and all data were coded into NVivo R1.7 by the lead researcher and checked by the senior researcher. A coding tree can be available on request. Final themes and sub-themes were agreed and then the analysis was presented in a narrative format. It was felt that data saturation was reached, as there were no new themes arising from the interviews and the key objectives were met. This qualitative study did not aim to provide generalisability to the patient population experiencing OC but to understand how acceptable a future VALTIVE2 trial was to study participants, as well as to understand their experiences of the trial and treatment.

Ongoing anonymised findings from interviews were regularly presented in real-time to the Study Management Team (SMT), which provided opportunities for feedback and reflections from the VALTIVE study team and PPI. Qualitative study participants were offered a summary of findings after completion of the analysis and the opportunity to provide feedback.

Reporting of our study is in accordance with the consolidated criteria for reporting qualitative research Consolidated criteria for Reporting Qualitative research checklist (online supplemental file 2).^19^

## Results

11 female participants took part in telephone interviews between September 2022 and October 2023. Participants were recruited from four sites. Interviews lasted between 19 and 69 min (32.5 min mean). Initially, twelve unaccompanied participants were interviewed; however, one participant was withdrawn due to an error in the main study’s recruitment process. The mean average age of ten participants was 62 years, median 65 years (range 50–71 years). These interview participants were a small self-selecting sample which did not include patients aged 75 years and over who represent around 28% of all new OC cases but did broadly compare to the average age of diagnosis between 60 and 67 years.^20^ All participants self-reported as white from British and Northern Irish backgrounds. No repeat interviews were carried out. Information about participants’ characteristics, including age, ethnicity, FIGO stage at diagnosis and treatment status at time of interview, is available in table 1.

**Table 1:** Participants characteristics

Participant	Age	Ethnicity	FIGO	Treatment status at time of interview
Participant 1	61–69	White	III	During treatment
Participant 2	61–69	White	II	Bevacizumab after completion of the cytotoxic chemotherapy
Participant 3	51–59	White	III	Post end of treatment
Participant 4	51–59	White	I	Bevacizumab after completion of the cytotoxic chemotherapy
Participant 5	61–69	White	III	Bevacizumab after completion of the cytotoxic chemotherapy
Participant 6	51–59	White	II	Bevacizumab after completion of the cytotoxic chemotherapy
Participant 7	51–59	White	III	Bevacizumab after completion of the cytotoxic chemotherapy
Participant 8	51–59	White	IIII	Post end of treatment
Participant 9	61–69	White	II	Bevacizumab after completion of the cytotoxic chemotherapy
Participant 10	Withdrawn			
Participant 11	71–79	White	II	On chemotherapy only, bevacizumab to follow
Participant 12	61–69	White	III	Post treatment (chemotherapy+bevacizumab)

FIGO, The International Federation of Gynaecology and Obstetrics.

The main findings are outlined as results of the inductive analysis, these include understanding of the study and implications, experiences of the study, and impact of treatments on quality of life. Additional themes reflect the objectives of the study, comprising the severity of side effects from bevacizumab; participants’ ability to differentiate between bevacizumab or other chemotherapy side effects; trade-offs between treatment and disease side effects in advanced disease; participants’ understanding of randomisation, and acceptability of treatment allocation where treatment may be discontinued. Selected quotations relating to these themes and sub-themes (table 2) are provided in the results, and all additional quotations are available in online supplemental file 3. The participants are denoted by a number, representing the order in which they participated.

**Table 2:** Qualitative themes and sub-themes

Themes	Sub-themes
1. Understanding of the VALTIVE1 study and implications	
2. Experiences of the VALTIVE1 study	2.1 Study burdens 2.2 Positive perceptions of VALTIVE1
3. Impact of treatments on quality of life	3.1 Changes to social life 3.2 Disruptions to daily life 3.3 Achieving hope and meaning in changed lives 3.4 Normalising side effects 3.5 Positive impact of treatments
4. The severity of side effects from bevacizumab	
5. Participants’ ability to differentiate between bevacizumab or other chemotherapy side effects	5.1 Side effects of chemotherapy 5.2 Side effects of bevacizumab
6. Trade-offs between treatment and disease side effects in advanced disease	6.1 Combined treatment side effects
7. Attitudes to VALTIVE2	7.1 Participant understanding of randomisation 7.2 Acceptability of treatment allocation where treatment may be discontinued 7.3 Preferences for knowing if bevacizumab was working 7.4 Timelines of bevacizumab

### Understanding of the VALTIVE1 study and implications

Participants interviewed in this study generally expressed satisfaction with the information and explanations provided to them before signing up to VALTIVE1.

It was made very clear to me … I had no … questions because everything was explained outright. (Participant 3)

While several participants understood its aims, some found it difficult to recall and were unclear about its purpose, indicating a need for checking participants’ understanding of the study and its treatments.

I’m not sure. I just think that probably they’re just going to use my bloods to find out how it reacts and things like that maybe. (Participant 1)

### Experiences of the VALTIVE1 study

#### VALTIVE1 study burdens

VALTIVE1 involved extra burdens for participants, including providing additional blood samples and paperwork. Despite this, participants were accepting of these additional tasks in return for contributing to the study and potentially receiving more monitoring. Several participants, however, expressed the need for better communication between the main treatment and local study teams. This included the need for blood samples and paperwork to be consistently coordinated with their main appointments, to reduce the need for extra appointments and travel that some participants had experienced. This was despite the study protocol stating extra appointments would not be required. Participants described a preference to be able to access phlebotomy services locally, and where this was experienced, they expressed gratitude.

Every time I got treated, I had to provide a blood sample for the trial … Also, I couldn’t because I got covid, so that delayed it, and then the next time the unit forgot to take the blood sample … which obviously, I was a bit disappointed with … Then they went and did it again, it’s just that I was more alert this time, because I happened to spot the vial and the paperwork that (Trials Officer’s name) had provided … So, I think there’s a bit of a failure there. (Participant 2)

#### Positive perceptions of VALTIVE1

Several participants described feeling positive about VALTIVE1 and felt that they had experienced no extra inconveniences.

I’m happy with everything. It’s not really made a difference to my life, as in hindrance, it’s just part of what I’m going through [laughs]. It’s just part of me story, really. (Participant 6)

### Impact of study and treatments on quality of life

#### Changes to social life

Participants reported how the treatments affected their quality of life physically, psychologically and socially. The side effects of treatment at times manifested as a reduction in their social activities, including seeing less of their relatives and friends and needing to adapt their daily routines. This was usually due to their increased clinical vulnerability and their diminished energy levels and immunity. As VALTIVE1 was a non-interventional study, these side effects would also have been experienced with standard treatment outside of the study.

So, it’s changed a lot for me, cos me kids won’t come to the house if they’ve got any slight colds, or coughs or owt [anything], but we did allow me a friend in, who would see me, and I ended up with tonsilitis, which made me very ill and I lost half a stone in weight … Yeah, I’ve gotta be very careful, just got no immune system. (Participant 11)

#### Disruptions to daily lives

Disruptions to participants’ daily lives, including changes to their routine activities and hobbies, were at times driven by fear of exacerbating the tumour or treatment side effects.

It’s stopped me doing lots of things really … I just have to do things a little bit at a time. If I’m cleaning or anything, I can do a little bit because I have to sit down for half an hour … I used to love going out walking and I can’t really do that anymore … I can go for a little walk, but then I have to rest (chuckles). It just wears me out. (Participant 8)

Treatment regimens and additional appointments for blood samples, recentred participants’ focus on their illness and reduced their ability to participate in their usual routines or make future plans.

I haven’t been out as much, [my] social life—I suppose has been on hold because of the chemo and having to go to (names cancer centre) every three weeks, and to go twice, cos you have to go for bloods one day and then go another day for your treatment, that takes up a lot of time as well. (Participant 12)

#### Achieving hope and meaning in changed lives

Limited side effects or a gradual improvement to their physical symptoms after treatment often enhanced the participants’ mood, and at times influenced a more positive outlook.

I’ve got to pace myself. But I’m feeling stronger each day and I’m getting more energy each day. I’m feeling well, I’m sleeping well. I’ve got a good appetite… I’m driving. (Participant 4)

The opportunity for treatment and VALTIVE1 participation had provided some participants with hope for a cure or extended life; others expressed a desire for normalcy in contrast to the vicissitudes they had endured.

The chemo’s all finished now, so I’m hoping that I’ll just go back to normal and I’ll be able to start to try and build up my stamina levels … So, yeah, getting out and about and everything … the joints of my fingers and everything … it’s bearable at the moment, but I’m hoping it’s not going to get any worse … Because I don’t think I’d be able to drive and everything … Once I’m off of it (bevacizumab), hopefully, it might … all go back to normal. (Participant 3)

Despite experiencing fundamental changes to their lives, participants often expressed a need to appreciate the balance between the opportunity for treatment and adjusting to the challenges it brings. Participants utilised varying coping strategies, including ‘living for the day’ and accepting limitations to their lifestyles, which were often conveyed through gratitude or stoicism.

It was a complete change, that you sort of think—we’ve got to live each day because I’ve been trying to sort of keep myself away from people, so that I don’t get infections and things … I’ve got to actually live my life as well now … You just think, well each day is a gift and just make the most of it. (Participant 4)

Participants acknowledged that individuals require clear and consistent yet differentiated information and support to be emotionally ready to come to terms with the disease and treatments.

It is about having information at the right time, and when you’re ready to accept, or to have that information and support … You’ve got to be open. We’ve all got to be open to learning … I’ve learnt about cancer. If I talk to someone else about it … It’s a wealth of information that I now hold about ovarian cancer. (Participant 9)

#### Normalising side effects of treatments

Side effects of chemotherapy and bevacizumab were regularly normalised or downplayed by participants, including those with more debilitating outcomes. This indicated a need for greater support for patients in dealing with the physical and psychological impact of these side effects.

Lack of appetite generally, tired, bit twingy, a few aches and pains, just really didn’t want to do anything for a day or two, but after that it wore off and I was absolutely fine … it’s a small price to pay [laughs], shall I say in the realm of things. (Participant 5)

#### Positive outcomes of treatments

Positive outcomes from the treatments were also described by several participants, including improved quality of life and reduced tumour growth. Participants described their physical improvements after treatment.

I’ve had two scans since I started the treatment … one of the scans was soon after I finished chemotherapy … that didn’t show any tumour growth and then I had another scan … three or four months into the inhibitor treatment [bevacizumab] … that’s actually going well, I think … It is the best thing since sliced bread; to be honest, I’m bouncing around like you would never think there was anything wrong with me. (Participant 2)

### Severity of side effects from bevacizumab

The severity of side effects from bevacizumab varied between participants and most participants perceived these as significant but tolerable. However, the combination and timing of the different treatments (chemotherapy and bevacizumab) was felt to have influenced the increased severity of the side effects, including an inability to heal.

What I would say is, it was a really difficult time to receive bevacizumab … because they wanted to run it in the sort of final chemo’s … Once I had bevacizumab, that’s when I then started to feel really poorly. I just I couldn’t reach my own feet; I couldn’t put shoes on; I couldn’t walk … I’m used to muscles feeling like they’ve been pulled … here and there, but nothing in comparison to … what I felt after taking that drug … Don’t get me wrong, absolutely, it wasn’t helping, but would it have been better on a standalone? … For instance, although I now know, obviously, my liver has a problem with things, I won’t be able to be given anything now … They gave that to me on chemo five … I couldn’t get my scars from my operation to heal … I couldn’t bend or do anything. [My] scab would float off in the bath. I had to stop washing then and actually try and wash in a different way. It was just awful. Plus, I was pulling muscles, all over my body … So, they stopped it. (Participant 9)

### Patients’ ability to differentiate between bevacizumab or other chemotherapy side effects

#### Side effects of chemotherapy

Participants generally felt they were able to distinguish between the side effects of chemotherapy and bevacizumab. Certainty was at times expressed about what side effects were caused by chemotherapy, as this treatment was started before bevacizumab. The most commonly reported chemotherapy side effects were neuropathy, pain, nose bleeds or nose running, hair loss, nausea, memory loss, rashes, fatigue and high blood pressure. It was felt that comorbidities exacerbated the side effects of the treatment.

I had my chemotherapy on a Wednesday, come the Friday, Saturday, Sunday, I’d feel nauseous, no appetite, a bit twingy pain wise, it would just last a couple of days and then that would go, so that was while I was on the chemo. I had bad mouth ulcers as well, but other than that, no, just a bit of fatigue. (Participant 5)

Oh, a bit groggy really … First … I’ve got arthritis anyway, but it definitely affects my joints, the treatment, and it’s made my blood pressure up … I get funny feelings in the bottom of my feet, like, in the mornings when I get up its … my foot, the bottom of my feet are very delicate. (Participant 8)

#### Side effects of bevacizumab

Common side effects attributed by participants to bevacizumab included stiffness, pain, fatigue, nose bleeding or running, brain fog and significant aching in muscles.

The only side-effects I had with the Beva was my gums, swelling really up and then going back down again. Mouth being sore … it was just the Beva, that all started, and it’s like nose bleeds with it, very minor nose bleeds, every time I’d blow, there were blood there. (Participant 11)

### Trade-offs between treatment and side effects in advanced disease

The time-consuming nature of the treatment and how it requires putting participants’ lives on hold, was perceived as a trade-off for receiving the treatments.

The only thing generally, obviously is being tied to all the treatments, I can’t really make plans to say—go away or anything like that, until things settle down, it’s difficult to plan if I want to go away for a few days or something like that. (Participant 5)

#### Combined treatment side effects

One participant felt that the side effects of the combined chemotherapy and bevacizumab were, in retrospect, not worth the potential gains they had offered.

I mean, it is a gamble. I know it’s percentages. You know, if the bevacizumab added two per cent more to your success rate, you know, was it worth having? And some people would say that two per cent was worth having, but I’m not sure. I would be one of these people that would say: ‘Is it though, if you can’t sleep at night, if you’re tired all the time? … You have to always ask yourself what you’re saving your life for.’ (Participant 9)

### Attitudes to VALTIVE2

A general explanation of the potential VALTIVE2 randomised trial was provided to the participant by the interviewer. Explaining that participants allocated to group A would receive standard treatment (chemotherapy and bevacizumab) only. Participants allocated to group B would receive standard treatment and blood tests at regular intervals, where the Tie2 concentration levels would be tested to detect vascular control in the tumour. Then, if vascular control was not detected, bevacizumab would be discontinued.

#### Participant understanding of randomisation

Varying levels of understanding of randomisation were reported by participants. Some understood the concept, while others required further explanation. Certain participants equated the ‘placebo effect’ with being randomised, as they perceived it as being allocated to a group receiving an intervention and one group that did not. While not strictly applicable to the potential VALTIVE2 trial, it demonstrated a broad understanding of being randomised.

So, one of us is going to be given some treatment and the other one is going to be, it’s going to be the placebo effect, isn’t it, go on? (Participant 2)

#### Acceptability of treatment allocation where treatment may be discontinued

Most participants felt that allocation to a group where bevacizumab may be discontinued based on the results from the biomarker test was acceptable, if not preferable. These participants stated a preference to be informed whether bevacizumab was working to prevent tumour growth.

If it’s stopped working, I mean, then there’s no point you know, carrying on. (Participant 3)

I think I’d prefer to know; I’d prefer to be into the group that I wanted to go into. (Participant 11)

One participant stipulated that if they were to participate in the randomised trial and thus allocated to either group, then it would be acceptable if they were monitored via regular CT scans to ensure that their condition was stable.

I would, I’d be happy to do that because they’re going to do CT scans on me every three months. (Participant 4)

#### Preferences for knowing if bevacizumab was working

Several participants’ preferences were motivated by concerns that continuing with bevacizumab when it was ineffective, that it would reduce their chances of trying alternative treatments and in turn their chances of survival or cure.

If it’s not working and … so they’re saying sort of after this drug, then potentially if you need something else, there’s another clinical trial … So, in some way, it could be positive, that if it’s not working, you’re not wasting time … If potentially, you can have something else that would be more effective. (Participant 4)

Unexpectedly, two study participants had already experienced the discontinuation of bevacizumab. Their attitudes were therefore informed by this experience, and they felt certain that they preferred to stop bevacizumab and, where possible, try an alternative treatment.

I think I’d prefer to do it how I did it before, having the blood test … No, it [bevacizumab] hasn’t [worked], because while I was on it, the cancer come active again, that’s why they stopped it … they’re on about putting me on a white pill. (Participant 11)

#### Timelines of bevacizumab

One participant felt that they may not want to discontinue treatment even where a test indicated that it was not working. They expressed concerns that the timelines for taking bevacizumab may be too short to be certain whether the treatment was or potentially could work in the future. This indicates the need to ensure participants are aware of the potential timescales of bevacizumab efficacy.

Because it’s not working at the beginning, would it better just to carry on till the end … so if I was to have this, the Avastin (bevacizumab) and then in August they say it’s not working how do I know, it would probably always play on my mind, well hang on a minute, officially was having it till April, what if in November it starts working [laughs]. I’d never know, would I? (Participant 6)

## Discussion

### Principal findings

VALTIVE1 participants reported commonly recognised side effects from bevacizumab and chemotherapy. The combination of treatments was felt to have influenced the severity of the side effects and exacerbated symptoms related to comorbidities. Ongoing information and support were required by participants regarding the study, treatment side effects and future expectations. Participants’ responses to the concept of the hypothetical future trial VALTIVE2 illustrated that most participants felt that it was acceptable, if not preferable, to be allocated to a group where bevacizumab may be discontinued based on the results from the biomarker test. They also conveyed a clear preference to be informed of the biomarker test results.

### Comparison to other literature

In this study, side effects attributed by participants to bevacizumab included stiffness, joint and muscle pain (arthralgia), fatigue and thrombocytopenia (low level of platelets) induced epistaxis (nose bleeding) and gingival (gum) bleeding. These findings are consistent with previously documented side-effect profiles in clinical trials.^21 22^ While most of these side effects were considered tolerable by participants, others, such as poor wound healing, were not. Participants’ perceptions of side effects are important to understand, particularly in relation to tolerability, as this can potentially impact study compliance and continuation.

Combination therapy has previously been associated with increased risk of poorly tolerated side effects such as gastrointestinal proliferation and thromboembolism (blood clots from another site in the circulatory system).^23^ This highlights the high level of risk that can be associated with these treatments and the potential impact on quality of life. However, participants in this study tended to normalise the side effects they experienced, including those with severe impact, reflecting prior studies, where side effects were often under-reported.^14 24^ This reticence to report side effects illustrates the need for greater awareness regarding expected side effects among patients and clinicians throughout the cancer pathway and improved access to support for symptom management and supportive care.^14 25^ Appropriate patient-centred communication is associated with better health-related quality of life and lower symptom burden among individuals with OC.^26 27^ Ongoing communication is required post-treatment, as side effects often continue.^28^ Participants highlighted their need for regular clinical updates and the opportunity to know the results of the biomarker tests.^29^,^31^

The physical, psychological, social and practical burdens of treatments on participants manifested as a reduction in their social activities, including seeing less of their relatives and friends and needing to adapt their daily routines and employment.^32^ Avoidable burdens were also a result of VALTIVE1, including additional appointments for blood samples, extra travel and paperwork usually caused by a lack of coordination between clinical services.^28^ There was a need to improve communication and consistency between clinical services which reflect patients’ prior experiences of OC care.^33^

### Strengths and limitations of this study

Strengths of this qualitative study are that it provides in-depth and nuanced participant experiences of receiving standard treatment for advanced OC in VALTIVE1. It illustrates the side effects and psychological impact of these treatments on patients and their attitudes towards bevacizumab discontinuation. The complexities of participants’ experiences are not captured through quantitative and clinical data. Few studies have implemented real-time reporting, which in this study provided ongoing analysis of data which was fed back to the SMT. This process supported the early identification of recruitment challenges in VALTIVE1 and opportunities to address them. Amendments to the consent to contact process were made to allow the recruitment team to send the consent to contact form on behalf of the participant to avoid delays in the qualitative team receiving their contact details. Additionally, real-time reporting highlighted potential improvements to VALTIVE2 before its implementation. This includes ensuring all future participants are provided with the opportunity to receive the results of the biomarker test, and the appointment of a centralised trial research nurse to support recruitment and improve participant and staff understanding of the trial. Particular attention will be given to the number of visits, including the blood sample collection schedule and reducing unnecessary paperwork. Public and patient representatives will ensure that patients’ perspectives are prioritised.

Limitations to the study include recruitment challenges. Significant delays in receiving essential permissions from VALTIVE1 sites were encountered by the qualitative study’s researchers between December 2021 and June 2022. These challenges resulted in a need to revisit the qualitative study’s original timeline, which was extended until October 2023. Participants were unable to be interviewed at baseline due to delays, so questions relating to recruitment processes were asked in interviews after treatment, which may have reduced participants’ ability to fully and accurately recall their experiences. The process of splitting the consent process between the initial consent to contact form and then the consent to the qualitative study created a more complex process for recruitment. Participants also consented separately to VALTIVE1 and the qualitative study. In some cases, the qualitative researchers were not sent appropriate paperwork, as described in the participant section. Consequently, several potential participants were not able to participate, despite this, data saturation was met.

### Future research and practice

Improvements to informed consent in a future trial could be facilitated through more user-friendly patient information materials and ensuring that trial and clinical staff check participants’ understanding of the trial. Additionally, participants should receive comprehensive information about the potential timescales of bevacizumab efficacy and the opportunities for alternatives if it is not effective. Ongoing support should be provided for symptom awareness and management post-treatment. The provision of regular updates on health status and test results could reduce patient anxiety and improve patient satisfaction. Measuring the influence of the timing of taking bevacizumab alongside chemotherapy and the wider impact of co-morbidities relating to the treatment outcomes could also be considered. Also, a high level of coordination and communication between the main treatment and trial sites is required to reduce extra travel and time burden on participants.

To ensure qualitative elements are adequately incorporated in future trials, an opt-out consent process for qualitative studies could be included in the consent form for the main trial. The simultaneous opening and consent of participants of the qualitative study and the main trial could be applied. This would allow for more time-efficient recruitment processes.

### Conclusion

Participants’ preferences for a future VALTIVE2 trial included being informed of whether bevacizumab was working and to discontinue bevacizumab if it had not prevented tumour growth based on the biomarker results. It should also consider measuring an individual’s treatment tolerability in relation to polypharmacy and comorbidities and ensuring that patients have comprehensive information and support regarding treatment side effects and expectations.

## Supplementary material

10.1136/bmjopen-2024-088474 online supplemental file 1

10.1136/bmjopen-2024-088474 online supplemental file 2

10.1136/bmjopen-2024-088474 online supplemental file 3

## Data Availability

All data relevant to the study are included in the article or uploaded as supplementary information. Data are available upon reasonable request.
