# Correction: Participants’ perspectives of the advanced ovarian cancer biomarker study VALTIVE1: a qualitative study

**DOI:** 10.1136/bmjopen-2024-088474corr1

**Published:** 2025-10-29

 Holland-Hart D, Carucci M, Slusarczyk M, *et al*. Participants’ perspectives of the advanced ovarian cancer biomarker study VALTIVE1: a qualitative study. *BMJ Open* 2025;15:e088474. doi:10.1136/bmjopen-2024-088474

This article was previously published with an error.

A retracted paper (reference 23) was cited in the Discussion section. This has now been deleted and the text has been updated from:

Combination therapy has previously been associated with increased risk of poorly tolerated side effects such as gastrointestinal proliferation, thromboembolism (blood clots from another site in the circulatory system), and reduction in wound healing.

to

Combination therapy has previously been associated with increased risk of poorly tolerated side effects such as gastrointestinal proliferation and thromboembolism (blood clots from another site in the circulatory system).

