# Supplementary material for: Participants’ perspectives of the advanced ovarian cancer biomarker study VALTIVE1: a qualitative study
Source: BMJ Open. 2025 Jul 13;15(7):e088474. doi: 10.1136/bmjopen-2024-088474 (PMC12258328; doi:10.1136/bmjopen-2024-088474)

**BEFORE THE INTERVIEW**

**Rapport building**

- E.g. Introduce self with some general background, Ask something about their local area

**Introduction**

- Thank you for agreeing to help me with this project – give a few details about the trial (e.g. name, centres, etc)
- I would also like to emphasise that only the study research team will see the information you give me.
- Your name will never be attached to any of the information you might share with me
- As I mentioned, I am going to record the conversation to allow us to analyse the interview in detail.
- However, in order for me to do this, I need to have your written consent
- Consenting the companion: As explained your …… is welcome to make any point or comment during the interview. However, again for us to be able to use the information given, we need to consent ……. as well.
- Last but not least, if anything is not clear, please stop me at any time
- State the purpose of the interview

**INTERVIEW GUIDE for participants at baseline**

**Today we are going to talk about how you have been feeling lately and how you feel about taking part in the trial. The interview should take between 30 and 60 minutes. If you need a break at any time, please let me know. Are you comfortable to carry on with the interview? (Reassure participants that this is not a test of knowledge – people remember different things)**

**Questions specific to VALTIVE**

1. When did you first hear about the trial?
   - 1. Who explained it to you?
     2. What information were you given?
     3. Was the information clear?
2. Can you tell me why you decided to participate in the trial?
   - 1. What were your main motives for joining?
     2. Did you feel supported when making this decision?
     3. Did you discuss the information given with others?
     4. Did you feel you had enough time to think about the information given?
3. Are you receiving treatment now?
4. How do you feel?

**Questions specific to VALTIVE2**

**SHOW VALTIVE2 PROMPT (see figure at the end of the document)**

**Give full explanation**

1. What is your understanding of the randomisation process in VALTIVE2?
   - - - 1. (Try to explore understanding of equipoise)
         2. (How they feel about uncertainty if raised)
2. If you were approached to take part in the VALTIVE2 study, how would you feel about being randomly allocated to group A or B? explain groups
3. If allocated to group B, you might be stopping one of the treatments (bevacizumab) if your blood tests show that is no longer effective for you. How would you feel about this?
4. Do you have any ideas about how we can give clear information to future participants to the trial?

**Concluding questions**

1. I have been asking you many questions, is there anything you would like to ask me?
2. Would you/how would you like to receive the results of this interview study?

**INTERVIEW GUIDE for participants during follow up**

**Today we are going to talk about how you have been feeling lately, your experience of the treatment you received and how you feel about taking part in the trial. The interview should take between 30 and 60 minutes. If you need a break at any time, please let me know. Are you comfortable to carry on with the interview? (Reassure participants that this is not a test of knowledge – people remember different things)**

**Specific questions**

**Joining the trial**

1. How have you found being part of a clinical trial so far?
   - 1. Anything negative or that could be improved?
     2. What are the positive aspects of the trial for you?
2. Have you had any questions or concerns since you started on the trial?
   - 1. Have you spoken to somebody about these concerns?
     2. Were your concerns addressed?

**Treatment**

1. Have you had all your treatment?
2. Have you been given any information on how the treatment is working for you? How it may have affected the current state of your illness?
   - 1. What was your reaction to this information?
     2. What did you think of the way the information was given to you?
3. How long have you been on your treatment?

**Questions specific to VALTIVE2**

**SHOW VALTIVE2 PROMPT (see figure at the end of the document)**

1. What is your understanding of the randomisation process in VALTIVE2?
   - - - 1. (Try to explore understanding of equipoise)
         2. (How they feel about uncertainty if raised)
2. If you were approached to take part in the VALTIVE2 study, how would you feel about being randomly allocated to group A or B?
3. How would you feel if you were allocated to group B and, following your last blood test, you were no longer receiving one of the treatments (bevacizumab) because it is no longer effective for you?
4. If this happened, with hindsight, would you have preferred to be randomly allocated to group A?

**Treatment experiences**

1. How have you been feeling?
   - 1. in yourself?
     2. symptoms/side effects?
     3. Coping (If there are psychological difficulties or coping difficulties, how have they been addressed? Has the participant talked to anyone? Who supports them?)
2. In reference to the symptoms you mentioned, how you manage them?

**Impact of treatment on quality of life**

1. How has your treatment affected your daily life?
   - 1. Has it stopped you from doing certain things?
     2. How have these symptoms affected your family and social life?
2. How is your quality of life since starting the treatment?
3. Does your treatment affect your family/social life?

**Impact of Coronavirus Pandemic (Note: only in the event that the pandemic is still ongoing and the patient has not mentioned it when answering the other questions).**

1. Do you feel that the coronavirus pandemic has had any impact on your treatment or recovery?
   - 1. How has the coronavirus pandemic affected your quality of life in relation to this trial?
     2. Has the coronavirus pandemic had an impact on your care or support during this trial?

**Accessing other services**

15. Have you been accessing other services? (eg Macmillan or Marie Curie)?

- - 1. Are there any other kinds of support you feel would benefit you?
    2. Would you know how to access these?

**Concluding questions**

1. I have been asking you many questions, is there anything you would like to ask me?
2. Would you/how would you like to receive the results of this interview study

**Potential VALTIVE 2 trial – Explanation Prompt**


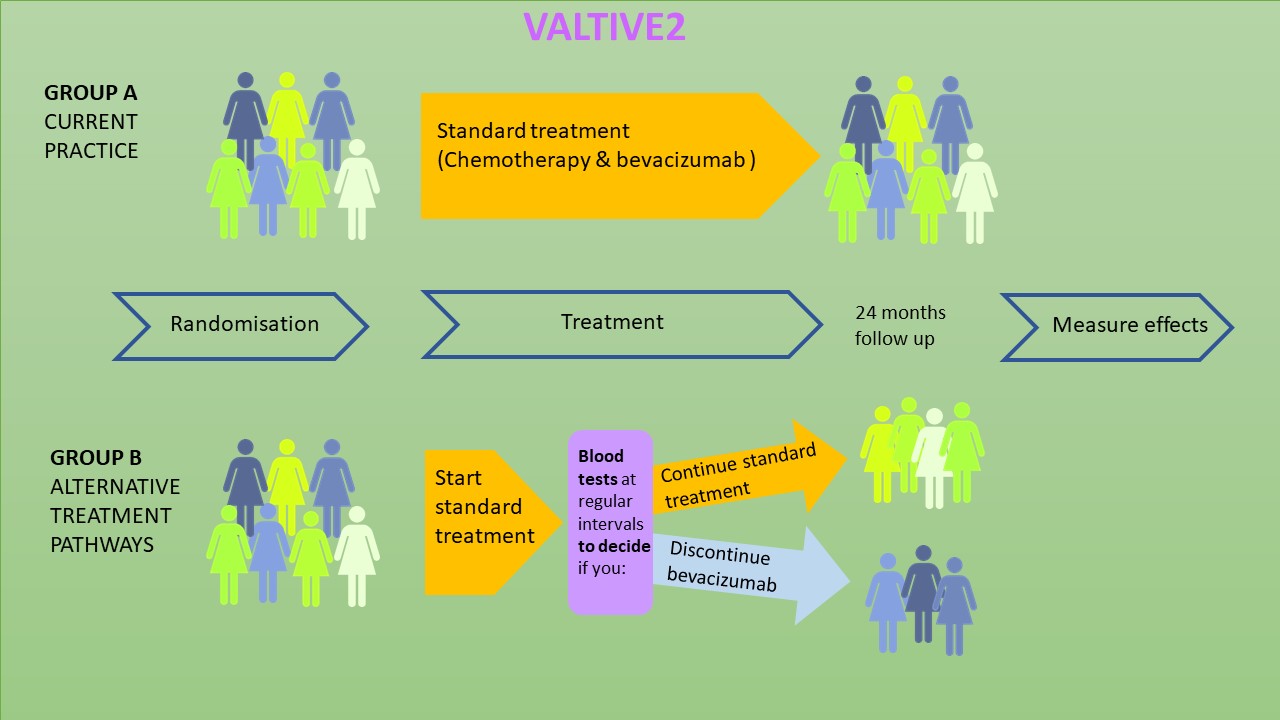

Supplement: online supplemental file 1 [file bmjopen-15-7-s001.docx]
