# Supplementary material for: Participants’ perspectives of the advanced ovarian cancer biomarker study VALTIVE1: a qualitative study
Source: BMJ Open. 2025 Jul 13;15(7):e088474. doi: 10.1136/bmjopen-2024-088474 (PMC12258328; doi:10.1136/bmjopen-2024-088474)
Supplement: online supplemental file 3 [file bmjopen-15-7-s003.docx]

| **Key Themes** | **Additional Quotations** |
| --- | --- |
| **1.Understanding of VALTIVE1 and implications** | |
| Participants interviewed in this study generally expressed satisfaction with the information and explanations provided to them before signing up to VALTIVE1. | So, I was aware what the study entailed… Is it to find out if it could be rolled out to other patients? (Participant 1)  I was quite happy with the information that I got… I know the treatment, obviously, has got approval, otherwise, I wouldn’t be having it. (Participant 2)  It was made very clear to me... I had no… questions, because everything was explained outright. (Participant 3)  It just really confirmed what was going through my head that it was a good thing and that. (Participant 5)  It was definitely explained to me well. (Participant 6) |
| Several participants understood its aims, but some found it difficult to recall and were unclear about its purpose, indicating a need for checking participants’ understanding of the study its treatments. | I’m not sure. I just think that probably they’re just going to use my bloods to find out how it reacts and things like that maybe. (Participant 1)  I got the impression it was to provide indicators I think about how well the treatment was working or not working on various people. (Participant 2)  I think, is it to do sort of like with the side effects of treatment and everything? (Participant 3)  I thought the aim of the trial was because in about 80% of the cases it’s a very effective drug to use. But whether they can, and it’s used as a first-line at the moment. They’re putting it, whether it can be used as a second-line. So, if ladies either to maintain, so that the cancer doesn’t develop or, or if it comes back. But at the moment, they can only use it once up to 18 infusions. (Participant 4)  Erm, well from what I read from all the information, it’s giving everybody all around, both the patients and consultants and doctors and medical professionals that better understanding of maybe what they can do to maybe tailor it to work better, just to maybe minimise people’s suffering as you go along the line. Maybe helping it not to get to a state where it’s too late, if you know what I mean. (Participant P 5)  The aim of the study… I’m guessing it’s for, well I shouldn’t be guessing, it’s for new medication for the future and to try and to, because I was at Stage 4 aggressive… to try and prevent that from happening to other ladies. (Participant 6)  What I understood is that, Avastin is given to lots of women, when they first, have ovarian cancer, [coughs], but for cost reasons and I guess cause it’s not always effective it’s not prescribed on the NHS, second time round, or third time round and the idea is to try to identify which group of women would benefit most, from being prescribed that drug… if there was recurrence. (Participant P 7)  I suppose just to make, see how it was working for me, for research purposes. (Participant 8)  I’m a bit fascinated, by the study, the idea that that the treatment is stopping these cells, setting up their own blood supply to create tumours. (Participant 12) |
|  | I’ve got to appreciate that one- I’ve been offered this Avastin for another 12 months and then two I’ve been offered these tablets for the next two years, because, you know, not everybody gets the chance to get this extra Avastin, normally it’s you’ve had your chemo, that’s… See you, not see you later, but you’re always going to be under their umbrella, aren’t you, probably for the five years or so. (Participant 6)  I didn’t know in the beginning how that drug would be administered. I didn’t quite maybe understand why we started it early. That’s, what I maybe had thoughts around, was: ‘How come we did it on, chemo five and six? You know, why was it started then?’… I wasn’t quite sure why that happened, why there was an overlap. (Participant 9) |
| **2. Experiences of the VALTIVE1 study** | |
| *2.1 VALTIVE1 Study burden* | |
| VALTIVE1 involved extra burdens for participants including providing additional blood samples and paperwork. Despite this, participants were accepting of these additional tasks in return for contributing to the study and potentially more monitoring. Several participants however, expressed the need for better communication between the main treatment and local study teams. This included the need for blood samples and paperwork to be consistently coordinated with their main appointments, in order to reduce the need for extra appointments and travel that some participants had experienced. This was despite the study protocol stating extra appointments would not be required. Participants described a preference to be able to access phlebotomy services locally and where this was experienced, they expressed gratitude. | The appointment’s come through, it was a bit tricky at the beginning though, I must admit… because I was getting conflicting appointments. Which was a bit bizarre, yeah. My blue card came with three dates on. And then I got an email from somebody to say it was a different time. They were all the same days but all different times. So, I did phone somebody and asked them ‘Which one am I doing?’ … And she apologised… and she did say ‘I think there’s been a bit of a mix-up because I think (Trust’s Name) are thinking the Research are getting in touch. And the Research are thinking that (Trust’s Name) are getting in touch’. (Participant 1)  Every time I got treated, I had to provide a blood sample for the trial… Also, I couldn’t because I got covid, so that delayed it, and then the next time the unit forgot to take the blood sample… which obviously, I was a bit disappointed with… Then they went and did it again, it’s just that I was more alert this time, because I happened to spot the vial and the paperwork that (Trials Officer’s name) had provided... So, I think there’s a bit of a failure there. (Participant 2)  The treatment and everything and other appointments are going round me; I’m having to write everything down… so that doesn’t clash. (Participant 3)  If I wasn’t on the trial, I would have been able to go to a local centre to have the treatment. … Which is about 15 minutes away but in order to be on the trial, I would have to go down to (Health Trust’s Name) for that. But I felt that was worth the sacrifice… It’s been a little bit difficult in some respects because my day is a Monday, which… on some occasions it’s a bank holiday… So, when I was due the first dose, the chemotherapy nurse said, ‘Oh we can’t take the bloods today because nobody’s available to test them’. So, I had the chemotherapy; I had all the treatments, but then I got a phone call from the trial people saying, ‘Can you pop down to have your bloods taken?’ So, of course, it’s, it’s a two-hour journey. For a five-minute blood test… I would have still said ‘Yes’ to the trial, but I wish they would have said, in the recruitment, that if it happens on a bank holiday, the possibility is, you’re going to have to come down another day… And only on one time did they have all the paperwork for me… I’ve had to take the paperwork with me, and the name of the person who’s overseeing the trial, because they said they haven’t got the paperwork with them. And they don’t know what bloods to take… Because they’ve said, ‘Well who do we call?... And they are literally just in another bay, just round the corner. On the third one, they’d already put the canula in, and they said ‘Oh I’ll have to take bloods from your other arm for that’ which is fine, but when you’re being told there’s no extra needles or anything, because I’m having quite a lot of blood tests and infusions… It was just an extra one… But she did manage to get the information but again, that slows you down then, because there was 10 or 15 minutes trying to sort that out… But I feel as if I’ve got to go armed ready to, to almost fight to get the bloods taken for the trial. (Participant 4)  I’ve been going to the out of hours services also getting me bloods took… nearer to home … rather than me going down to [hospital name]… they run like a clinic at, it’s like at a hospice, … and they also provide the chemotherapy there and do the bloods… So, a few times I’ve said to them, “oh are you taking the bloods for the trials as well”? and then they say, “oh no!”, and it’s as if they don’t really know about it… So, I just go, take me bloods and presume that everybody’s interlinked with each other and… they take my blood, because I was told … it was 11 extra blood tests… I have queried it a couple of occasions… making sure that am I also getting me trials bloods took at the same time to save them doing it again... Hopefully I can just keep going to nearer to home which would be easier for us, personally, … I go where I’m told to go basically, at the minute. I don’t want to mess anybody up. (Participant 6)  You come out with loads of appointments, and they’re all ..., you’re hitting those a few times a week. (Participant 9)  If I could do the bloods locally… because it’s part of a trial, I don’t know if they would agree to that. I did ask the last time… I was at the (names cancer centre) the ward, the treatment centre was very busy, and they never got an answer back by the time I’d finished my treatment. (Participant 12) |
| *2.2 Positive perception of VALTIVE1* | |
| Several participants described feeling positive about VALTIVE1 and felt that they had experienced no extra inconveniences. | Having the drugs is part and parcel to sort of keeping me going, so I’m quite happy to take part in it, but it’s not affected me in anyway at all… it’s not taking any extra time or any extra effort on my part so providing an extra sample, having an extra test, having an extra scan... I’m not being asked to do an awful lot extra than what I am doing anyway, so I don’t see the problem with it, to be honest. (Participant 5)  I’m happy with everything. It’s not really made a difference to my life, as in hindrance, it’s just part of what I’m going through [laughs]. It’s just part of me story, really. (Participant 6)  It’s just blood tests and that’s it really… it hasn’t really affected me … not the actual… study. (Participant 9) |
| **3. Impact of treatments on quality of life** | |
| *3.1 Changes to social life* | |
| Participants reported how the treatments affected their quality of life physically, psychologically and socially. The side-effects of treatment at times manifested as a reduction in their social activities, including seeing less of their relatives and friends and needing to adapt their daily routines. This was usually due to their increased clinical vulnerability and their diminished energy levels and immunity. As VALTIVE1 was a non-interventional study, these side-effects would also have been experienced with standard treatment outside of the study. | So, it’s changed a lot for me, cos me kids won’t come to the house if they’ve got any slight colds, or coughs or owt [anything], but we did allow me friend in, who would see me, and I ended up with tonsilitis, which made me very ill and I lost half a stone in weight…Yeah, I’ve gotta be very careful, just got no immune system. (Participant P11)  I think it’s affected everything really... Of course, I do have a social life but… not so much. (Participant 8).  I pop into [Name of place], we’re like a little village, town and everything… And I’m going out sort of like to shop and everything… but I mean I must admit I do like the old comfort now, Sainsbury’s actually delivering to me rather than going to pick it up. (Participant 3)  I’m just being careful not to be in crowded places really. (Participant 4) |
| *3.2 Disruptions to participants’ daily lives* | |
| Disruptions to participants’ daily lives including changes to their routine activities and hobbies were at times driven by fear of exacerbating the tumour or the side-effects of treatments. | I mean, there are a few things that I don’t do… I don’t go to the gym on a regular basis… whereas I used to before, but I couldn’t after I had the cancer, because I had a lung drain put in and it wouldn’t allow me to do anything, well, basically, I wasn’t allowed to play sports for quite some time. (Participant 2).  It’s stopped me doing lots of things really… I just have to do things a little bit at a time. If I’m cleaning or anything, I can do a little bit because I have to sit down for half an hour … I used to love going out walking and I can't really do that anymore… I can go for a little walk, but then I have to rest (chuckles). It just wears me out. (Participant 8)  I’m a lot weaker, I can’t do what I used to do, I used to be quite fit, I used to do all the gardening, my housework, everything, the house is suffering a bit, me oldest son helps out with gardening, [I’m] definitely weaker, and I daren’t try and cos, I’m frightened, I might aggravate the tumour. (Participant 11) |
| Treatment regimens and additional appointments for blood samples, recentred participants’ focus on their illness and reduced their ability to participate in their usual routines or make future plans. | I think the other big thing that’s sort of impacting is, because you’re having the infusions every three weeks, but you don’t know whether, if your blood pressure is high or you’ve got protein in your urine or anything it’s going to be delayed. You can’t plan for any holidays or anything… Everything’s got to be very last minute. Which is fine, but I’m normally a very organised person so, it’s taking a bit to get my head round… it’s just sort of you have to plan your life very differently. (Participant 4)  To be fair I haven’t got a big and busy social life anyway, so I can’t really say it had that big an impact… the only thing generally… is being tied to all the treatments, I can’t really make plans to say, go away or anything like that, until things settle down, it’s difficult to plan if I want to go away for a few days or something like that, but, I’m hoping now… this particular drug and the chemo has actually finished that I will be able to slot in maybe the odd few days away here and there, so long as things stay on plan. (Participant 5)  I haven’t been out as much, [my] social life - I suppose has been on hold because of the chemo and having to go to [names cancer centre] every three weeks, and to go twice, cos you have to go for bloods one day and then go another day for your treatment, that takes up a lot of time as well. (Participant 12) |
| *3.3 Achieving hope and meaning in changed lives* | |
| Limited side-effects or a gradual improvement to their physical symptoms after treatment often enhanced the participants’ mood, and at times influenced a more positive outlook. | It’s actually going well, I would say relatively speaking, my energy levels are very good. (Participant 2)  I mean I’m quite glad that I’ve not had any sickness or anything like that... I used to do a lot of walking and everything and that... I’m starting to do a bit more walking but not what I used to be able to and everything, because obviously now with this joint issue and everything that I’m suffering with and everything. (Participant 3)  I’ve got to pace myself. But I’m feeling stronger each day and I’m getting more energy each day. I’m feeling well, I’m sleeping well. I’ve got a good appetite…I’m driving. (Participant 4)  I’ve been going on long walks, we do like kind of five mile walks quite regularly and I’ve been going on my exercise bike, and I’ve been feeling really well to be honest… yeah, until last week when I started developing the symptoms. (Participant 7)  I’m back to normal again... I feel fine at the minute. I ... walked ten miles on Friday. I have no trouble doing that. I don’t ache afterwards, I can still move around. I do six very often, just to go and get my coffee. So, I still lift weights in my own house. I’m not, lifting a stupid amount of weights – I’m aware that my body’s been into surgery and been into things – but I do my own stuff. (Participant 9)  I think I’ve been very blessed… I haven’t had the sickness and everything that a lot of people suffer, and I had this three ... lots of chemo to reduce the cancer and then had a big operation. I recovered very well from that, and then I’ve continued the chemo, so I feel as though I’ve been very lucky really in that way. (Participant 12) |
| The opportunity for treatment and VALTIVE1 participation had provided some participants with hope for a cure or extended life; others expressed a desire for normalcy in contrast to the vicissitudes they had endured. | I’ve been told twice they’re going to cure me, so that’s keeping me going. (Laughs). (Participant 1)  The chemo’s all finished now, so I’m hoping that I’ll just go back to normal and I’ll be able to start to try and build up my stamina levels… So, yeah, getting out and about and everything… the joints of my fingers and everything… it’s bearable at the moment, but I’m hoping it’s not going to get any worse… Because I don’t think I’d be able to drive and everything… Once I’m off of it (bevacizumab), hopefully, it might… all go back to normal. (Participant 3)  It was disappointing… I suppose I was almost thinking… potentially next September, the treatments finish and hopefully I can have a period of normality... I feel positive because I think being positive and aiming is good… if the treatment is effective… We’ve heard of people doing very well on, on this drug and for several years. (Participant 4)  I want to go back to work… but it’s not been an easy ride at home… it’s been a bit same old, same old and this is why I need to get back to it, because I want to feel normal again… I’ll probably never feel normal, I’ll always feel… my life will never be what it was because of what I’ve been through and stuff, and what if it comes back… and… what if I start getting cancer again… I’m going to live my life like that, but, so yeah, at the minute, mentally I’m fine. I’m feeling a bit low as in, oh I’m sick of it now, I just want it all to be normal but it’s not going to happen. (Participant 6) |
| Despite experiencing fundamental changes to their lives, participants often expressed a need to appreciate the balance between the opportunity for treatment and adjusting to challenges it brings. Participants utilised varying coping strategies including ‘living for the day’ and accepting limitations to their lifestyles, which were often conveyed through gratitude or stoicism. | The thing is, I know when I do too much. If I get tired or think, I only do little bits. So, as long as I feel I’ve done something, it makes me feel better. (Participant 1)  So, is it my destiny to have it kind of thing… so, a bit morbid… but at the moment… I’m hoping that nothing will happen in the future and everything. (Participant 3)  It was a complete change, that you sort of think, we’ve got to live each day because I’ve been trying to sort of keep myself away from people so that I don’t get infections and things… I’ve got to actually live my life as well now… You just think, well each day is a gift and, and just make the most of it. (Participant 4)  It’s not necessarily a cure – I’ve got still cancer in me right now. I’m not cured, I don’t ring a bell. It’s not gone. It’s not going anywhere, ever. So, I have to get used to the new me and the new world I move in, and that expands and expands. The further into it I get, the easier it seems, the more confident I become, the more confidence is given to me by the people I see… and that ... the horizon moves. But initially, that horizon’s way too close to be told everything... I can’t look into next year, and I can’t plan five years now, and I don’t know whether I’ll be sixty… People have to learn to try and live with what’s going on around them and learn how to adjust it and make it work for them – and this works for me. (Participant 9)  I’ve got this cancer, that’s not gonna be able to kill it off, they just wanna try and stabilise it, I know all that, I think just live for each day and hope they can stabilise it and it’ll be okay for a while. (Participant 11) |
| Participants acknowledged that each individual requires clear and consistent yet differentiated information and support to be emotionally ready to come to terms with the disease and treatments. | When I first went to see the Oncologist, he was saying they were aiming for a cure and that what I hadn’t appreciated was, when going on the maintenance drug… they’d found some cancer cells over my liver and over my bowel… I still thought they were going for a cure, but when I asked the consultant if we were still going for a cure, he said ‘No, now we’re going to control it so for as long as the drugs work. But there are other drugs as well, that we can use after the Avastin’. I suppose, I didn’t understand that when the clinician phoned me with the results of the CT scan, because she said that and I said, ‘Oh that doesn’t sound good’. And she said ‘Oh, no, it’s fine’… So, I was thinking that we were still going for a cure. So, I suppose it was a little bit of a shock. (Participant 4)  It is about having information at the right time, and when you’re ready to accept, or to have that information and support… You’ve got to be open. We’ve all got to be open to learning... I’ve learnt about cancer. If I talk to someone else about it ... It’s a wealth of information that I now hold about ovarian cancer. (Participant 9) |
| *3.4 Normalising side-effects of treatments* | |
| Side-effects of chemotherapy and bevacizumab were regularly normalised or downplayed by participants including those with more extreme and debilitating outcomes. This indicated a need for greater support for patients in dealing with the physical and psychological impact of these side-effects. | Obviously, hair loss, but that was expected. It was coming out, so I just got my hairdresser, she popped round and shaved it all off. (Participant 1)  [My] side effects, minor, I think have an issue with my nose, it doesn’t generally bleed, occasionally, I will get spotting, but… that’s… not very often., I think my brain cells are under, under functioning, but that could be old age… I think I’m just getting a bit more forgetful about things… as I said to people, “Well, you know, they pump that much poison into you, something is bound to go wrong! (Participant 2)  I lost a bit of hair, because even though I had a cold cap on, I didn’t lose a lot of it… My Oncologist… said ‘Are you happy to go on?’ I said ‘Yeah’, I mean I’ve only got six more sessions apparently still to go and… well… as it stands at the moment…. I’ve been quite fortunate, quite lucky and everything with not having many side effects. These are the only couple of like side effects that I’ve kind of like, and a bit of brain fog as well. (Participant 3)  Lack of appetite generally, tired, bit twingy, a few aches and pains, just really didn’t want to do anything for a day or two so, but after that it wore off and I was absolutely fine… it’s a small price to pay [laughs], shall I say in, in the realm of things. (Participant 5)  The chemo was not too bad to be honest… I had bad joint pain which I think was caused by Taxol for maybe five days, but apart from that I had quite a lot of energy and I’ve, yeah been well, really generally. (Participant 7)  I had the usual chemo problems: really bad joints; waking up every eight minutes. I might get an eight-minute break, fall asleep and then I’m awake again, in pain and misery, the usual stuff that we all get. (Participant 9) |
| *3.5 Positive benefits of treatments*  Positive benefits from the treatments were also described by several participants, including improved quality of life and reduced tumour growth. Participants described their physical improvements after treatment. | I’ve had two scans since I started the treatment… one of the scans was soon after I finished chemotherapy... that didn’t show any tumour growth and then I had another scan… three or four months into the inhibitor treatment [bevacizumab]…, that’s actually going, well, I think ... It is the best thing since sliced bread: to be honest, I’m bouncing around, like you would never think there was anything wrong with me. (Participant 2)  My two chemo’s… I think they’re gold standard… My cancer was making me very ill: wetting myself, pain and misery. You know, taking painkillers all the time... I was going to the toilet to pee a tiny amount, literally round the clock... because the tumours were so big, pushing on the lines… between my kidneys and my bladder… So, as soon as I had chemo, I felt better… on the first one... On the second one, I felt better again. You know, I actually noticed a difference on my first three, running up to the operation. I felt awful after the operation, but that ... That’s different… again. (Participant 9) |
| **4. The severity of side effects from bevacizumab.** | |
| The severity of side-effects from bevacizumab varied between participants, and most participants perceived these as significant but tolerable. However, the combination and timing of the different treatments (chemotherapy and bevacizumab) was felt to have influenced the increased severity of the side-effects including an ability to heal. | [It’s] been very painful and everything and then trying to sort of like get out of bed to stand up, it’s like, you know, I feel like I’m a 90-year-old and everything, you know. [Participant 3]  I felt like me legs felt a little bit heavy, like, oh, you know, a bit dull achy, at the bottom… I think because I had the Avastin (bevacizumab). (Participant 6)  What I would say is, it was a really difficult time to receive bevacizumab… because they wanted to run it in the sort of final chemo’s … Once I had bevacizumab, that’s when I then started to feel really poorly. I just I couldn’t reach my own feet; I couldn’t put shoes on; I couldn’t walk… I’m used to muscles feeling like they’ve been pulled ... here and there, but nothing in comparison to ... what I felt after taking that drug... Don’t get me wrong, absolutely, it wasn’t helping, but would it have been better on a standalone?... For instance, although I now know, obviously, my liver has a problem with things, I won’t be able to be given anything now… They gave that to me on chemo five... I couldn’t get my scars from my operation to heal… I couldn’t bend or do anything. [My] scab would float off in the bath. I had to stop washing then and actually try and wash in a different way. It was just awful. Plus, I was pulling muscles, all over my body… So, they stopped it. (Participant 9) |
| **5. Patients’ ability to differentiate between bevacizumab or other chemotherapy side-effects.** | |
| *5.1 Side-effects of chemotherapy* | |
| Participants were generally able to distinguish between the side-effects of chemotherapy and bevacizumab. Certainty was at times expressed about what side-effects were caused by chemotherapy, as this treatment was started before bevacizumab. The most commonly reported chemotherapy side-effects were neuropathy, pain, nosebleeds or nose running, hair loss, nausea, memory loss, rashes, fatigue and high blood pressure. It was felt that comorbidities exacerbated the side-effects of the treatment. | I had my chemotherapy on a Wednesday, come the Friday, Saturday, Sunday, I’d feel nauseous, no appetite, a bit twingey pain wise, it would just last a couple of days and then that would go, so that was while I was on the chemo. I had bad mouth ulcers as well, but other than that, no, just a bit of fatigue. (Participant 5)  I had bad joint pain which I think was caused by Taxol. (Participant 7)  Oh, a bit groggy really… First … I’ve got arthritis anyway, but it definitely affects my joints, the treatment, and it’s made my blood pressure up … I get funny feelings in the bottom of my feet, like, in the mornings when I get up its … my foot, the bottom of my feet are very delicate. (Participant 8)  Chemo, obviously, I lost my hair… I used to have nose bleeds. They weren’t bad nose bleeds... I did manage to get on my feet, after chemos, but obviously, you kind of crash and burn a bit, you fall off a cliff a bit as the drugs settles in you. I had rashing… which I took lots of antihistamines for. I managed to work my way past that by not touching my skin, not getting anything hot, not rubbing it with anything, you know, scented, or anything unusual… I had the usual chemo problems: really bad joints; waking up every eight minutes… I might get an eight-minute break – you know, fall asleep – and then I’m awake again, in pain and misery. You know, the usual stuff that we all get. Constipation, really bad. You know, there’s a lot of howling at the moon and crawling on the floor and crying a lot. (Participant 9) |
| *5.2 Side-effects of bevacizumab* | |
| Common side-effects attributed by participants to bevacizumab included stiffness, pain, fatigue, nose bleeding or running, brain fog and significant aching in muscles. | So, when I came off the paclitaxel and the carboplatin and then was just on the bevacizumab… This was when I started getting side-effects on its own and everything, you know, sort of like, in the mornings, trying to move my hands… I mean these are the only couple of like side-effects that I’ve kind of like, and a bit of brain fog as well. (Participant 3)  I’ve been kind of blowing my nose quite a bit, it’s like that constant… kind of runny nose which I know is one of the side effects of, of Avastin. (Participant 7)  The only side-effects I had with the Beva was my gums, swelling really up and then going back down again. Mouth being sore… it was just the Beva, that all started, and it’s like nose bleeds with it, very minor nose bleeds, every time I’d blow, there were blood there. (Participant 11) |
| **6. Trade-offs between treatment and side-effects in advanced disease** | |
| The time-consuming nature of the treatment and how it requires putting participants’ lives on hold, was perceived as a trade-off of receiving the treatments. | The only thing generally, obviously is being tied to all the treatments, I can’t really make plans to say- go away or anything like that, until things settle down, it’s difficult to plan if I want to go away for a few days or something like that. (Participant 5) |
| *6.1 Combined treatment side-effects* | |
| One participant felt that the side-effects of the combined chemotherapy and bevacizumab was in retrospect not worth the potential gains they had offered. | I mean, it is a gamble. I know it’s percentages. You know, if the bevacizumab added two per cent more to your success rate, you know, was it worth having? And some people would say that two per cent was worth having, but I’m not sure. I would be one of these people that would say: ‘Is it, though, if you can’t sleep at night, if you’re tired all the time?... You have to always ask yourself what you’re saving your life for. (Participant 9) |
| **Attitudes to VALTIVE2** | |
| *7.1 Participant understanding of randomisation* | |
| Varying levels of understanding of randomisation were reported by participants. Some understood the concept, whilst others required some explanation. Certain participants equated the ‘placebo effect’ with being randomised, as they perceived it as being allocated to a group receiving an intervention and one group that did not. Whilst not strictly applicable to the potential VALTIVE2 trial, it demonstrated a broad understanding of being randomised. | Because if you’re random and you don’t know. (Participant 1)  So, one of us is going to be given some treatment and the other one is going to be, it’s going to be the placebo effect, isn’t it, go on? (Participant 2)  Interview: Do you have any understanding of the randomisation process?  Participant: Yes, yes I do. (Participant 4)  One set is given the real thing and the other’s given like a placebo type thing, is that what you mean? (Participant 5)  Do you mean the, the canula in me arm, do you mean, is that what you mean? (P6)  So, what you’re saying is there’s one group which is random and the other group is, if there’s evidence that, the patient is responding well and would benefit from carrying on, am I right? (Participant 7)  Interview: So, what we’re trying to find out is whether, what your understanding of the randomisation process is? I, don’t know if you know that is?  Participants: No. No. (Participant 8) |
| *7.2. Acceptability of treatment allocation where treatment may be discontinued* | |
| Most participants felt that allocation to a group where bevacizumab may be discontinued based upon the results from the biomarker test was acceptable, if not preferable. These participants stated a preference to be informed whether bevacizumab was working to prevent tumour growth. | I think I’d like to know if it was stopped… And if it was to stop, I’d accept that result. (Participant 1)  Oh, I’d like to know about it. (Participant 8)  If it’s stopped working, I mean, then there’s no point you know, carrying on. (Participant 3)  I think I’d prefer to know, I’d prefer to be into the group that I wanted to go into. (Participant 11)  Hopefully find it is working or not? (Participant 12) |
| One participant stipulated that if they were to participate in the randomised trial and thus allocated to either group, then it would be acceptable if they were monitored via regular CT scans to ensure that their condition was stable. | I would, I’d be happy to do that because they’re going to do CT scans on me every three months. (Participant 4) |
| *7.3 Preferences for knowing if bevacizumab was working* | |
| Several participants’ preferences were motivated by concerns that by continuing with bevacizumab when it was ineffective, that it would reduce their chances of trying alternative treatments and in turn their chances of survival or cure. | Well, that (randomisation) wouldn’t worry me… so, being taken off of it, because it wasn’t working… I will just sort of think, well what’s the point of continuing with it, if it’s not working? (Participant 2)  Yeah. I think, I would be happy to do it. I suppose it’s just you don’t want to think that it’s not working, do you? (Participant 4) |
|  | I think I would be a little (concerned) because I mean, the situation that I am in at the moment... And I’m just thinking well, I’m on bev at the moment and now I’ve been told that two of my tumours have sort of like grown… and I’m thinking… I thought bev is supposed to stop like the vessels growing, so how are these growing, kind of thing? (Participant 3)  If it’s not working and… so they’re saying sort of after this drug, then potentially if you need something else, there’s another clinical trial… So, in some way, it could be positive, that if it’s not working, you’re not wasting time… If potentially, you can have something else that would be more effective (Participant 4) |
| Unexpectedly, two study participants had already experienced the discontinuation of bevacizumab. Their attitudes were therefore informed by this experience and these participants felt certain that they preferred to stop bevacizumab and where possible try an alternative treatment. | If, with what’s been happening to me in the past week… it makes things quite difficult… if… my CA-125 levels had remained low all the way through then I would probably be happy to go ahead with this but… because it, it’s not looking very good at the moment, I’m worried that it would jeopardise my chances of getting a different treatment. (Participant 7)  I think I’d prefer to do it how I did it before, having the blood test… No, it [bevacizumab] hasn’t [worked], because while I was on it, the cancer come active again, that’s why they stopped it… they’re on about putting me on a white pill. (Participant 11)  Everyone can make the choice to go on it, and everybody would have it taken away if they made a complaint. (Participant 9) |
| *7.4 Timelines for bevacizumab* | |
| One participant felt that they may not want to discontinue treatment even where a test indicated that it was not working. They expressed concerns that the timelines for taking bevacizumab may be too short to be certain whether the treatment was or potentially could work in the future. Indicating the need to ensure participants are aware of the potential timescales of bevacizumab efficacy. | Because it’s not working at the beginning, would it better just to carry on till the end… so if I was to have this, the Avastin (bevacizumab) and then in August they say it’s not working how do I know, it would probably always play on my mind, well hang on a minute, officially was having it till April, what if in November it starts working [laughs]. I’d never know, would I? (Participant 6) |
