# Supplementary material for: Participants’ perspectives of the advanced ovarian cancer biomarker study VALTIVE1: a qualitative study
Source: BMJ Open. 2025 Jul 13;15(7):e088474. doi: 10.1136/bmjopen-2024-088474 (PMC12258328; doi:10.1136/bmjopen-2024-088474)
Supplement: Supplementary file 4 [file bmjopen-2024-088474.reviewer_comments.pdf]

PEER REVIEW HISTORY

BMJ Open publishes all reviews undertaken for accepted manuscripts. Reviewers are asked to complete a checklist review form and are provided with free text boxes to elaborate on their assessment. These free text comments are reproduced below.

ARTICLE DETAILS

Title (Provisional)

Participants' perspectives of the advanced ovarian cancer biomarker study VALTIVE1: a qualitative study.

Authors

Holland-Hart, Daniella; Carucci, Margherita; Slusarczyk, Magdalena; Longo, Mirella; Campbell, Susan; Irving, Alys; Noble, S; Jayson, Gordon; Hopewell-Kelly, Noreen

---

VERSION 1 - REVIEW

**Reviewer**	**1**
**Name**	**Biskup, Edyta**
**Affiliation**	**Department of Pathology, Copenhagen University Hospital**
**Date**	**24-Jul-2024**
**COI**	**None**

Manuscript by Holland-Hart et al entitled "Participants' perspectives of the study and treatments of (VALTIVE1) an advanced ovarian cancer biomarker study" presents results of phone interviews with 11 patients enrolled in VALTIVE1 study. Healthcare workers and scientists should remember that ultimately patients are those who are in the center of a given trial and thus patients' perspective should be taken under consideration, and their voice heard. The manuscript in question also underscores the significance of patients' being well-informed about the outome of the treatment (biomarker test results) and involved in the clinical decisions (possible discontinuing of the bevacizumab). In this respect, I think the manuscript is important and should be published.

I have only few comments/questions

Page 8. Choice of participants. In the Methods it is stated that participants were recruited from four out of nineteen sites recruiting to VALTIVE1. Why only four and why these four? Can the Authors say anything more about the structure of the group? (age range and median, stage distribution, was English their first language or can the results also be influence by the language barrier?)

Page 9. Delays. It says that most participants were contacted by the study team several months after consenting to the main study. Could it affect the outcome? (for examle, casually speaking, could the patient have forgotten ”how badly it felt”? Or was the time of the interview itself not affected? Also, the baseline interviews (before the treatment) are being mentioned. It is a bit unclear to me: were the baseline interviews still conducted before the treatment, despite the delays?

Page 12. Participants expressed satisfaction with the information and explanations provided. Yes, it is important to inform patients about the treatment so they understand how it is going to be performed and what to expect. If the patients were happy with the way the information was provided, can the Authors also inform how it was done? Was the information provided orally or written? If the latter, was it a plain-language information leaflet? Could the patients at any time receive clarification if something was not clear or they have forgotten some inportant pieces?

Figure. A number and proper title plus description is missing. Also, I understand the meaning of this figure, but I would consider a slight modification showing that originally we are dealing with one cohort and randomisation results in a split into groups A and B.

Eventually, I understand that the study in question is a qualitative study and I do not expect detailed statistical analysis (which would be anyway unrealistic with a group of 11 patients). However, I was wondering, if it wouldn’t be possible to add a table/fuigure summarizing prevalence of certain outcome/feelings. I think it would allow readers a betterg grasp of Authors’ findings. For example, how many (out of 11) reported certain outcome, based on themes summarized in Table 1.

Minor comments

I would consider modifying the title slightly, since ”Participants’ perspectives of the study and treatments of (…) study” sounds a little off to me. Maybe rather: ”Participants’ perspectives of VALTIVE1 - an advanced ovarian cancer biomarker study” or ”(…) – a biomarker study in advanced ovarian cancer”

Page 8. Capital ”P” in Participant Information Sheet is missing

Page 16. ”Positive benefits” – I don’t think it’s a correct pharasing, benefits cannot possibly be negative.

Page 20. I am not sure if a given example shows that the concept was fully understood (the placebo effect). Generally, placebo can only be used if no treatment for a certain condition is given. Here, we compare a standard-of-care with an improved treatment (plus bevacizumab). However, I understand that it might be a matter of discussion what the placebo effect means in this context (bevacizumab vs. no bevacizumab).

Page 34. Concluding questions shouldn’t be point 6 if Specific questions is not numbered, I think

Page 37 Why are points 11 and 12 suddenly between 6 and 7?

**Reviewer**	**2**
**Name**	**Guo, Xiaoyue Mona**
**Affiliation**	**USC Keck School of Medicine**
**Date**	**05-Oct-2024**
**COI**	**None**

This is a well written article describing the qualitative component of a larger biomarker study evaluating the utility of the Tie2 protein in determining response to bevacizumab for advanced ovarian cancer patients. The qualitative portion assesses patient acceptability to obtaining an additional lab test and potentially stopping treatment based on it. The study personnel should be commended for their effort in ensuring a patient-centered study that takes patient experiences and perspectives into consideration. While a small study sample, they found that participants were able to tease out the bevacizumab-specific adverse effects, and importantly, would accept discontinuation of the drug based on biomarker testing. I would recommend a few additions/clarifications about the underlying study and the study population, as I came away confused about what VALTIVE1 involves.

METHODS:

- Please provide either a short summary of the VALTIVE1 study protocol or link to a prior protocol paper if available. There is a study schema at the end of the paper for VALTIVE 2, can you provide that for VALTIVE1 as well? What were the inclusion/exclusion criteria – was this for primary ovarian cancer patients? Also, is it typical for all advanced staged ovarian cancer patients to receive bevacizumab concurrently with platinum-based therapy? Does this change by institution?

- Can you clarify if all patients in VALTIVE1 were approached for the qualitative study? What was the recruitment rate and how representative of the full population are those who agreed to be interviewed?

- When were patients interviewed – during or after their chemo? If after, how long after?

RESULTS:

- Please provide a table with more patient demographics than just the age – both of the entire VALTIVE 1 population and the 10 patients who agreed to interview. Stage and histology of cancer, chemotherapy obtained/completed, BRCA status, use of concurrent Parp, how many cycles, prior medical history that could have affected side effect profile, etc

- You mention in 2.1 that VALTIVE1 includes extra burdens for participants – Could you quantify this burden? How many more visits, how much blood and paperwork was exactly required? Again, having the protocol provided will help.

DISCUSSION:

- How much of the physico-psycho-social burden is due to pariticpation of the study vs obtaining chemotherapy?

- Did you ask patients how much was the burdens due to the extra appts and paperwork for the study specifically?

- Do the results of this study change how real-time PROs will be handled in VALTIVE2?

- Large limitation is the small number of patients included, and whether their experiences can generalize to the entire population.

**Reviewer**	**3**
**Name**	**Miyamoto, Morikazu**
**Affiliation**	**Department of Obstetrics and Gynecology, National Defense Medical College Hospital, Department of Obstetrics and Gynecology**
**Date**	**27-Jan-2025**
**COI**	**No**

This paper is participants' perspectives of the study and treatments of (VALTIVE1) an advanced ovarian cancer biomarker study. This paper was not the study to analyze anything. Therefore, I considered, because it was not appropriate for the research study, it was rejected.

**Reviewer**	**4**
**Name**	**Starkings, Rachel**
**Affiliation**	**Brighton and Sussex Medical School, SHORE-C**
**Date**	**25-Mar-2025**
**COI**	**I have no competing interests to declare**

This is a well written and thought out piece of research. With the advent of tests which help personalize care, we need to ensure we provide robust qualitative work to demonstrate how patients feel and engage with these. There were some minor typographical errors to flag - in the second sentence of the first page you cite a source twice, in the recruitment section you say, 'with a planned completion date is August 2025' and in data collection you say the qualitative researchers have experience with qualitative research.

Would you be able to provide some information about how many people were approached for a qualitative interview? I note you do have the breakdown of why people declined but it would be great to know the figures for the wider pool. Given your recruitment process, I

understand you may not have this so perhaps include the number of consent to contact forms received.

You say there were 2 topic guides - one for those at baseline and one for post-treatment. It would be useful to understand a bit more information about the study to know how long people are followed up for and at which timepoint(s) you were trying to interview people. Understanding the timeframe you had to offer an interview would also add some clarity in understanding the impact of missing letters of access. I appreciate you don't want to report anything ahead of a main study publication but as the reader won't yet be able to access the background material, I think some detail should be included.

In your supplementary material, you include a topic guide for people who declined study but there's no comment made about this. Can you clarify?

Can you please provide further detail in the manuscript about your qualitative process. I appreciate the inclusion of the COREQ checklist but it would be a struggle to replicate your process from the checklist and manuscript as they are now. I think you can give an overview of the process from immersion to thematic development as I had questions about how the initial codes were agreed between you, how you practically managed the double coding, how discrepancies were identified and managed. Did you use Kappa, did you have an arbitrator? How did you handle the process of thematic mapping?

In the COREQ checklist, you note that interviews were conducted over the telephone but there's a diagram that you showed to people to explain the VALTIVE2 design. How did this work?

VERSION 1 - AUTHOR RESPONSE

**Reviewer 1**	**Author's responses**
Page 8. Choice of participants. In the Methods it is stated that participants were recruited from four out of nineteen sites recruiting to VALTIVE1. Why only four and why these four? Can the Authors say anything more about the structure of the group? (age range and median, stage distribution, was English their first language or can the results also be influence by the language barrier?)	Thank you for this question, we have now clarified that it is four out of the five sites that signed up to the qualitative study, of the nineteen studies that signed up to VALTIVE1. The data collection explains the challenges to recruiting participants and the difficulties in receiving permissions to individual sites.<br><br>The results sections now includes information about ethnicity. Information about whether English was their first language was not collected. However, translators/ interpreters were provided to patients on request. The age is also now described as a median, as well as mean averages, as follows:<br>'The mean average age of ten participants was 62 years, median 65 years (range 50-71 years), we were unable to locate the age of

	one participant. All participants self-reported as white from British and Northern Irish backgrounds.'
Page 9. Delays. It says that most participants were contacted by the study team several months after consenting to the main study. Could it affect the outcome? (for examle, casually speaking, could the patient have forgotten "how badly it felt"? Or was the time of the interview itself not affected? Also, the baseline interviews (before the treatment) are being mentioned. It is a bit unclear to me: were the baseline interviews still conducted before the treatment, despite the delays?	This is an important point and has now been addressed. Baseline interviews were not conducted after treatment but questions relevant to participants' experiences at the beginning of the trial were asked in the interviews after treatment. Further details have been added to the data collection section as follows:<br>'Participants were unable to be interviewed at baseline (before treatment), so they were asked questions about being recruited to the study during interviews after treatment started.'<br>It is also addressed in the strengths and limitations section:<br>'Participants were unable to be interviewed at baseline due to delays, so questions relating to recruitment processes were asked in interviews after treatment, which may have reduced participants' capacity to fully and accurately recall their experiences'.
Page 12. Participants expressed satisfaction with the information and explanations provided. Yes, it is important to inform patients about the treatment so they understand how it is going to be performed and what to expect. If the patients were happy with the way the information was provided, can the Authors also inform how it was done? Was the information provided orally or written? If the latter, was it a plain-language information leaflet? Could the patients at any time receive clarification if something was not clear or they have forgotten some inportant pieces?	The main trial did not collect information about how the study was explained to individual participants. However, additional information has been added to the text in the recruitment section to explain what information was provided and how it was explained:<br>'Potential participants were provided with information about the VALTIVE1 study and the qualitative study, via written participant information sheets, accompanied by an oral explanation provided by recruiting teams. All trial staff received site initiation training, which also covered how to explain the VALTIVE1 and the qualitative study to participants. The PIS was written in plain language and checked by the PPI representative to ensure it was user-friendly for participants. VALTIVE1 participants were provided with contact details of the trial staff in case they required further information about the study. Language translators and interpreters were provided to patients on request.'
Figure. A number and proper title plus description is missing. Also, I understand the meaning of this figure, but I would consider a	The figure mentioned, seems to be the figure included in the interview schedules. The description of the title has now been changed

slight modification showing that originally we are dealing with one cohort and randomisation results in a split into groups A and B.	to: Potential VALTIVE 2 trial – Explanation Prompt and an explanation of how it was used in the data collection section, as follows:<br>'This includes a visual representation prompt, viewed by the interviewer to help explain the potential randomised control VALTIVE2 trial to participants.'
Eventually, I understand that the study in question is a qualitative study and I do not expect detailed statistical analysis (which would be anyway unrealistic with a group of 11 patients). However, I was wondering, if it wouldn't be possible to add a table/fuigure summarizing prevalence of certain outcome/feelings. I think it would allow readers a betterg grasp of Authors' findings. For example, how many (out of 11) reported certain outcome, based on themes summarized in Table 1.	We have now emphasised the qualitative methodology in the title.<br>Thank for your suggestion about quantifying participants' experiences. However, due to the nuance and personalised nature of the qualitative accounts, it is not appropriate to quantify and over-generalise the outcomes and feelings of the participants. In additional to the illustrative quotations in the main paper, the additional quotations available in supplement 2 illustrate the range of outcomes and feelings that were expressed in relation to these comments.
Minor comments	
I would consider modifying the title slightly, since "Participants' perspectives of the study and treatments of (...) study" sounds a little off to me. Maybe rather: "Participants' perspectives of VALTIVE1 - an advanced ovarian cancer biomarker study" or "(...) – a biomarker study in advanced ovarian cancer"	This has now been modified in accordance with the suggestion follows:<br>'Participants' perspectives of the study and treatments of the advanced ovarian cancer biomarker study (VALTIVE1): a qualitative study'.
Page 8. Capital "P" in Participant Information Sheet is missing	This has now been amended.
Page 16. "Positive benefits" – I don't think it's a correct pharasing, benefits cannot possibly be negative.	This has now been amended to positive 'outcomes' from treatments...
Page 20. I am not sure if a given example shows that the concept was fully understood (the placebo effect). Generally, placebo can only be used if no treatment for a certain condition is given. Here, we compare a standard-of-care with an improved treatment (plus bevacizumab). However, I understand that it might be a matter of discussion what the placebo effect means in this context (bevacizumab vs. no bevacizumab).	Thank you for this comment. We have now provided more explanation of VALTIVE2 and how the participants understood randomisation, as follows:<br><br>'A general explanation of the potential VALTIVE2 randomised trial was provided to the participant by the interviewer. Explaining that participants allocated to Group A would receive standard treatment (chemotherapy and bevacizumab) only. Participants allocated to Group B would receive standard treatment and blood tests at regular interviews, where the Tie2 concentration levels would be tested to detect vascular control in the tumour. Then if vascular control

	was not detected, bevacizumab would be discontinued.'<br>In the randomisation section, it now discusses the understanding of randomisation in more detail, as follows:<br>'Varying levels of understanding of randomisation were reported by participants. Some fully understood the concept, whilst others required some initial explanation. Certain participants equated the 'placebo effect' with being randomised, as they perceived it as being allocated to a group receiving an intervention and one group that did not. Whilst not strictly applicable to the potential VALTIVE2 trial, it demonstrated a broad understanding of being randomised'.
Page 34. Concluding questions shouldn't be point 6 if Specific questions is not numbered, I think	Numbering for the interview schedule has now been amended.
Page 37 Why are points 11 and 12 suddenly between 6 and 7?	Numbering for the interview schedule has now been amended.
**Reviewer: 2**	
Please provide either a short summary of the VALTIVE1 study protocol or link to a prior protocol paper if available. There is a study schema at the end of the paper for VALTIVE 2, can you provide that for VALTIVE1 as well? What were the inclusion/exclusion criteria – was this for primary ovarian cancer patients? Also, is it typical for all advanced staged ovarian cancer patients to receive bevacizumab concurrently with platinum-based therapy? Does this change by institution?	There is now a reference to the protocol which was published in October 2024.<br>The participants section stated:<br>'Participants were aged 16 years or older and had FIGO stage IIIc/ IV ovarian cancer on treatment with first-line platinum-based chemotherapy and bevacizumab.'<br>Additional information has been provided in the background section regarding VALTIVE1 and how it only involves standard treatment for this specific stage of ovarian cancer, as follows:<br>'This non-intervention study involves standard treatment doses and schedules of bevacizumab. VALTIVE1 only incorporates extra plasma sample acquisition (at the same time as bevacizumab is administered) and monitoring of the patient's condition'.<br><br>Also, there is currently no trial schema for VALTIVE2, the diagram is an illustration of what a potential trial would look like and therefore was used to enquire what participants felt about the concept of a VALTIVE2 trial, a description of this is now included in the data collection section, as follows:

	'This includes a visual representation prompt, viewed by the interviewer to help explain the potential randomised control VALTIVE2 trial to participants.'
- Can you clarify if all patients in VALTIVE1 were approached for the qualitative study? What was the recruitment rate and how representative of the full population are those who agreed to be interviewed?	Information was not collected about the recruitment rate for the qualitative study but it did achieve its sample size. The recruitment section now includes more information about who was recruited, as follows:<br>'A sub-set of VALTIVE1 participants were invited to participate in interviews, with the aim of recruiting 10-20 participants'.
- When were patients interviewed – during or after their chemo? If after, how long after?	A table is now available in the results section, highlighting at what point during VALTIVE1 that participants were interviewed.
RESULTS:	
- Please provide a table with more patient demographics than just the age – both of the entire VALTIVE 1 population and the 10 patients who agreed to interview. Stage and histology of cancer, chemotherapy obtained/completed, BRCA status, use of concurrent Parp, how many cycles, prior medical history that could have affected side effect profile, etc	The VALTIVE1 study has not yet been completed and demographics for the main study will be published when the study is complete. Additional demographic information has been provided in the results section of this paper including ethnicity data, FIGO stage and treatment stage at time of interview but it would not be appropriate to provide any further level of detail including medical history etc. for the small sample interviewed in this qualitative study. This data will be provided when the VALTIVE1 study is published.
- You mention in 2.1 that VALTIVE1 includes extra burdens for participants – Could you quantify this burden? How many more visits, how much blood and paperwork was exactly required? Again, having the protocol provided will help.	A reference to the protocol is now available in the background section. Blood samples were provided in accordance with whether participants require up front surgery or chemotherapy. Therefore, there is no overall standard number of times that blood samples were required for VALTIVE1. The blood samples schedule is available in the published protocol. This is now mentioned in the background section:<br>'The blood sample schedule is available in the published protocol.'<br><br>Also, the qualitative data (section 2.1) provides examples of where participants were occasionally asked to provide duplicate or extra blood samples in cases where scheduled sample collection were missed, as well as those scheduled due to mistakes. The paperwork is described in the recruitment section as PIS, consent and

	consent to contact forms, which are required for the VALTIVE1 and the qualitative study.
DISCUSSION:	
- How much of the physico-psycho-social burden is due to pariticpation of the study vs obtaining chemotherapy?	The text currently outlines where participants are discussing the extra burdens of the VALTIVE1 study itself i.e. section 2.1 and in contrast the burdens of treatments, which is classed as standard care (sections 3,4,5,6) .<br><br>Participants were asked about the impact of treatment (relating to physical and psychological quality of life- i.e. questions 8-12 in the interview schedule) but not specifically on the separate impact of chemotherapy or bevacizumab.<br>Questions 1 and 2 asked about their general experiences of the trial.<br>The quotations associated with each answer illustrate the physical, psychological and social perspectives through the words and perspectives of participants. In some instances, the participants provide clear understanding and demarcation between how the different treatments and participation of the study impact these factors and provide a rationale e.g. chemotherapy began before bevacizumab started. These side-effects reflect the side-effect profile found in prior studies and referenced in the discussion section. However, as chemotherapy and bevacizumab were provided around the same time, it would be difficult to provide an exact distinction between the side-effects profiles for the two treatments, and this is why more general questions were asked about their overall how the treatment impacted on their physical/ psychological well-being and quality of life.
- Did you ask patients how much was the burdens due to the extra appts and paperwork for the study specifically?	Questions 1 and 2 in the interviews enquired about participants general experiences of the trial. Participants themselves highlighted (usually unprompted), the difference in how the extra appointments, blood tests and paperwork (planned or unplanned) were an extra burden or not.
- Do the results of this study change how real-time PROs will be handled in VALTIVE2?	More information has been provided in the strengths and limitations section, as follows: 'Particular attention will be given to the number of visits, and blood sample collection schedule, reducing unnecessary

	paperwork. Public and patient representatives will ensure that patients' perspectives are prioritised.'
- Large limitation is the small number of patients included, and whether their experiences can generalize to the entire population.	This qualitative study did not aim to provide generalisability to the whole population but to understand how acceptable a future VALTIVE2 trial was to study participants, as well as understanding their experiences of the trial and treatment, which is explained in the aims section, as follows:<br>'The aim of this qualitative interview study within VALTIVE1 was to inform the design of the future randomised trial (VALTIVE2). It sought to explore nuanced participants' experiences of the VALTIVE1 study and treatments, and how these impacted on their quality of life, rather than generalisability for those experiencing ovarian cancer. This included exploring how they might feel if their treatment were to be discontinued upon the results of a biomarker test'.<br>The recruitment section also now states that: 'The target sample size was initially 10-20 participants', This number was able to be achieved despite recruitment challenges.
**Reviewer 4**	
You say there were 2 topic guides - one for those at baseline and one for post-treatment. It would be useful to understand a bit more information about the study to know how long people are followed up for and at which timepoint(s) you were trying to interview people. Understanding the timeframe you had to offer an interview would also add some clarity in understanding the impact of missing letters of access. I appreciate you don't want to report anything ahead of a main study publication but as the reader won't yet be able to access the background material, I think some detail should be included.	Information has now been added to clarify the timeframe that the qualitative team were able to offer an interview for, in the Recruitment section, as follows:<br>'Once participants consented to VALTIVE1 and the qualitative study, they could be interviewed at any time point during their participation in VALTIVE1.'<br>The Recruitment and Data Collection sections also explain that due to delays in recruitment and permissions from clinical sites participants were not interviewed at baseline but asked questions during or after treatment.
In your supplementary material, you include a topic guide for people who declined study but there's no comment made about this. Can you clarify?	The supplementary materials have now been amended to illustrate the topic guides without the questions about those who declined the study, as this part of the proposed study was not included in the final protocol.
Can you please provide further detail in the manuscript about your qualitative process. I appreciate the inclusion of the COREQ checklist but it would be a struggle to replicate your process from the checklist and	In the data analysis section there is a description of the process of thematic analysis, to confirm how an agreement on codes and themes were reached more

manuscript as they are now. I think you can give an overview of the process from immersion to thematic development as I had questions about how the initial codes were agreed between you, how you practically managed the double coding, how discrepancies were identified and managed. Did you use Kappa, did you have an arbitrator? How did you handle the process of thematic mapping?	information has been added to the following paragraph:<br><br>'Data were analysed using both deductive and thematic inductive analysis. All the transcripts were coded by the lead author and 50% were double coded by the second author using NVivo R1.7. Initially, broad themes were captured by analysing interview data deductively, (16) and key findings were developed in accordance with the qualitative study's objectives described previously. Then an inductive thematic analysis (17) was conducted, focusing on developing a thematic framework. Themes were generated by combining relevant codes and identifying overarching concepts. Initially, researchers coded transcripts separately, then agreed a coding structure together, as well as the main themes and their concomitant categories. The coding structure and analysis framework was refined as an iterative process final codes and themes were agreed through ongoing deliberations. A codebook was then established to organise the themes and subthemes. Themes and sub-themes were agreed and then analysis was presented in a narrative format. It was felt that data saturation was reached, as there were no new themes arising from the interviews and the key objectives were met'.<br><br>Quantitative metrics such as Kappa were not used to measure and demonstrate a level of agreement for the coding and analysis, and an arbitrator was not used to decide on the final codes as agreement through ongoing deliberations was reached between the two researchers regarding the final framework. As described- NVivo was used to help manage the thematic mapping, after initial coding was conducted and a framework was created.
In the COREQ checklist, you note that interviews were conducted over the telephone but there's a diagram that you showed to people to explain the VALTIVE2 design. How did this work?	During the interview, the qualitative interviewers described the hypothetical VALTIVE2 study to the patient. The visual depiction of the VALTIVE2 study was used as an aid to describe the hypothetical VALTIVE2 trial. This detail has now been added to the data collection section.

Would you be able to provide some information about how many people were approached for a qualitative interview? I note you do have the breakdown of why people declined but it would be great to know the figures for the wider pool. Given your recruitment process, I understand you may not have this so perhaps include the number of consent to contact forms received.	Information is provided in the recruitment and results section about those who had initially consented or signed a consent to contact form from the four sites with appropriate which were available for recruitment into the qualitative study. However, information was not collected centrally about all participants who were approached for the study,

VERSION 2 - REVIEW

Reviewer 1

Name Biskup, Edyta

Affiliation Department of Pathology, Copenhagen University Hospital

Date 14-May-2025

COI

I have now read the modified version of the manuscript and I am satisfied with the changes Authors made. Thank you for your thorough work. I have only few rather cosmetic suggestions.

Page 8 – since a new paragraph has been added to the Recruitment, the ”PIS” abbreviation is now explained at the second, not first occurence

Table 1 – is there any reason for giving FIGO stages as Arabic rather than Roman numerals?

Quotations – in most cases the quotation is followed by the: ”Participant X” though there is at least one occurence of ”P X” (P 3; slight inconsistency)

Title. I understand that the Authors will stick to the edited title: ”Participants’ perspectives of the advanced ovarian cancer biomarker study (VALTIVE1): a qualitative study.” and I agree with that phrasing. However in the rebuttal (table) it reads: ”Participants’ perspectives of the study and treatments of the advanced ovarian cancer biomarker study (VALTIVE1): a qualitative study”, which does not sound too fortunate. But I guess it was a just mistake.

Reviewer 4

Name Starkings, Rachel

Affiliation Brighton and Sussex Medical School, SHORE-C

Date 30-May-2025

COI

Thank you for considering the revisions suggested. Overall I think the paper reads better and again I think it's great to be demonstrating patient engagement in future project development. I just have a few remaining points for your consideration.

I appreciate you submitted the COREQ checklist but I think it would be better to have all information in the paper and use the checklist to reference the location of this. In particular, I think the methodology in the manuscript is missing some reflexive elements, such as who you are and your relationship to participants. I do appreciate you say you have expertise carrying out this work, and there's no doubting that, but I do think you should be explicit to say you were all post-doctoral researchers with 'x amount' of experience in conducting interviews. I think the paper should also include your response to checklist question 6.

Thank you for your additional clarity around your qualitative process. I think this just needs some final clarity. As it reads now, it seems the deductive coding was done followed by inductive and then your coding structure was agreed. I think you just need to add a small bit of text to clarify whether the deductive and inductive coding were done all in one go, or whether you did these sequentially. If so, were both phases double-coded? You say you agreed the coding structure together - was there any double coding done after this agreement to confirm its use?

There were just a couple of typos – beginning paragraph on page 10 where you list the reasons for non-participation. I think there should be a comma between interview and three to continue the list rather than starting a separate sentence.

Recruitment – the first sentence should have an 'of' instead of 'is'.

VERSION 2 - AUTHOR RESPONSE

**Reviewer 1**	
Page 8 – since a new paragraph has been added to the Recruitment, the ”PIS” abbreviation is now explained at the second, not first occurence	The explanation for PIS has now been moved to the first mention of it, in the recruitment section.
Table 1 – is there any reason for giving FIGO stages as Arabic rather than Roman numerals?	The international federation that develops this system, called FIGO (International Federation of Gynaecology and Obstetrics), uses roman numerals. This paper is consistent with this form of numbering.
Quotations – in most cases the quotation is followed by the: ”Participant X” though there is at least one occurence of ”P X” (P 3; slight inconsistency)	This reference has now been changed from P3 to Participant 3.
Title. I understand that the Authors will stick to the edited title: ”Participants’ perspectives of the advanced ovarian cancer biomarker	We confirm the correct title is the Participants’ perspectives of the advanced ovarian cancer biomarker study (VALTIVE1): a

study (VALTIVE1): a qualitative study." and I agree with that phrasing. However, in the rebuttal (table) it reads: "Participants' perspectives of the study and treatments of the advanced ovarian cancer biomarker study (VALTIVE1): a qualitative study", which does not sound too fortunate. But I guess it was a just mistake	qualitative study. It was mistake written in the responses to reviewer's form.
**Reviewer: 4**	
I appreciate you submitted the COREQ checklist but I think it would be better to have all information in the paper and use the checklist to reference the location of this. In particular, I think the methodology in the manuscript is missing some reflexive elements, such as who you are and your relationship to participants. I do appreciate you say you have expertise carrying out this work, and there's no doubting that, but I do think you should be explicit to say you were all post-doctoral researchers with 'x amount' of experience in conducting interviews. I think the paper should also include your response to checklist question 6.	We have now added any relevant details into the main text, including in relation to question 6 . Page numbers are also added to the COREQ checklist.<br>As suggested, further detail has been added to the data collection element explaining are post-doctoral researchers with over ten years of experience each of interviewing people.<br>The paragraph has now been amended to the following:<br>'Semi-structured interviews were conducted by telephone by two female qualitative post-doctoral researchers (lead and senior authors). They have over ten years each of experience of exploring participants' trial experiences, and sensitive health related topics including cancer. The qualitative researchers had no prior relationship to the participants and used their research experience and training in qualitative methods to introduce the research study and mitigate the asymmetry of information between the two parties. They were not involved in participants' treatment or any other part of the main study, and therefore, were able to critically explore elements of participants' experiences of VALTIVE1. Participants were made aware of the researchers' roles, university and department where they were based and the purpose of the study before the interview.'
Thank you for your additional clarity around your qualitative process. I think this just needs some final clarity. As it reads now, it seems the deductive coding was done followed by inductive and then your coding structure was agreed. I think you just need to add a small bit of text to clarify whether the deductive and inductive coding were done all in one go, or whether you did these sequentially. If so, were both phases double-coded? You say you agreed the coding	This paragraph has been amended to further clarify the process including that 50% of the transcripts were originally double coded by the senior author. The term 'synchronously, has been added to the sentence 'Synchronously, an inductive thematic analysis was conducted' to demonstrate that coding was done together.<br>Then, the paragraph goes on to state that all of the main themes and sub-themes were agreed on through an iterative process

structure together - was there any double coding done after this agreement to confirm its use?	agreed with both researchers and that the themes were coded into NVivo by the lead author and checked by the senior author, as follows:<br>'All the transcripts were originally coded by the lead author and 50% were double coded by the second senior author using NVivo R1.7. Initially, broad themes were captured by analysing interview data deductively, and key findings were developed in accordance with the qualitative study's objectives. [17] Synchronously, an inductive thematic analysis was conducted. [18] Themes were generated by combining relevant codes and identifying overarching concepts. Initially, researchers coded transcripts separately, then agreed a coding structure together, including all the main themes and their concomitant categories. The coding structure and analysis framework was refined as an iterative process, final codes and themes were agreed through ongoing deliberations. A codebook was then established to organise the themes and subthemes, which both researchers agreed on and all data was coded into NVivo R1.7 by the lead researcher and checked by the senior researcher. Final themes and sub-themes were agreed and then analysis was presented in a narrative format.
There were just a couple of typos – beginning paragraph on page 10 where you list the reasons for non-participation. I think there should be a comma between interview and three to continue the list rather than starting a separate sentence.	The manuscript has been further checked for typos and these have been amended.
Recruitment – the first sentence should have an 'of' instead of 'is'.	This has now been amended.
